# Influence of Cleft Laterality on Cleft Width, Canine and Lateral Incisor Status, and Adjacent Midfacial Midline Structures: A Retrospective CBCT Analysis

**DOI:** 10.3390/diagnostics16193140

**Published:** 2026-09-27

**Authors:** Hanadi Sabban

**Affiliations:** Department of Oral Diagnostic Sciences, Division of Oral and Maxillofacial Radiology, Faculty of Dentistry, King Abdulaziz University, Jeddah 21589, Saudi Arabia; hsabban@kau.edu.sa

**Keywords:** cone beam computed tomography, cleft palate, cleft lip, nasopalatine canal, nasal septum, dentoalveolar anomalies, alveolar cleft, cleft laterality

## Abstract

**Background/Objectives**: This retrospective study examined, in patients with cleft palate, whether canine eruption status is associated with lateral incisor status, whether cleft width differs by laterality, age, or sex, and whether nasopalatine canal and septal position correlate with cleft laterality. **Methods**: CBCT examinations of 33 patients were reviewed, including small subgroups of 7 unilateral right-sided, 14 unilateral left-sided and 12 bilateral cases. Lateral incisor and canine status were compared using Fisher’s exact test; cleft width was compared by laterality, age, and sex using the Kruskal– Wallis, Spearman, and Mann–Whitney U tests; and canal and septal position were tested against laterality using the Fisher–Freeman–Halton test, with comparisons repeated excluding the six youngest patients. **Results**: Canine eruption status was not associated with lateral incisor status (right *p* = 1.000; left *p* = 0.733). Root resorption occurred in 13.2% of present lateral incisors, and at least one was absent in 45.5% of patients (all *p* > 0.14 for age, sex, and side). Canine eruption status was associated with age (unerupted canines younger, 10.8 vs. 15.1 years; *p* = 0.005). Cleft width did not differ by laterality, age, or sex (all *p* > 0.14; ICC 0.974). Canal and septal position correlated significantly with laterality (*p* = 0.002, *p* = 0.001): the canal deviated away from the cleft side in 87.5% of unilateral cases, and the septum toward it in 90.5%; findings were unchanged after excluding the youngest patients. Intra-examiner reliability for these two variables was almost perfect (Cohen’s κ = 0.86–0.88). **Conclusions**: Dentoalveolar findings showed no significant association with cleft laterality, apart from the expected age-eruption link, and should be read as inconclusive given the modest sample size. Canal and septal position, by contrast, were significantly and reciprocally correlated with laterality, consistent with cleft palate reflecting a regional craniofacial disturbance rather than an isolated alveolar defect, relevant to pre-surgical planning pending larger-cohort confirmation.

## 1. Introduction

Orofacial clefts, encompassing cleft lip, cleft palate, and combined cleft lip and palate, are among the most common congenital craniofacial malformations. A recent systematic review and meta-analysis placed the global prevalence at 0.45 per 1000 live births, with figures across individual regional studies commonly falling between approximately 1 in 500 and 1 in 700 live births [1]. Mossey [2] emphasized the value of international, multi-center birth-defects registries for establishing consistent epidemiological associations across populations with differing genetic and environmental backgrounds. Data pooled from the related International Perinatal Database of Typical Oral Clefts (IPDTOC) demonstrate a six-fold variation in the birth prevalence of cleft lip with or without cleft palate across populations and a three-fold variation for isolated cleft palate [3], and regional reviews focused on Arab populations report an average orofacial-cleft prevalence of approximately 1.1 per 1000 live births, with cleft palate identified as the predominant subtype in several countries of the region [4]. Beyond the facial and nasal deformity, itself, orofacial clefts are consistently associated with a constellation of Dento-alveolar disturbances in the cleft region, including tooth agenesis, malformed or displaced teeth, altered eruption sequences, and structural anomalies of the alveolar bone that together complicate long-term orthodontic and surgical rehabilitation [5].

The alveolar cleft is a bony defect of the maxillary alveolar process that normally houses the roots of the lateral incisor and canine, and it is present in the majority of patients with cleft lip and/or palate [1]. Because this defect can disturb Dento-alveolar growth, tooth eruption, and periodontal support in the adjacent dentition, accurate three-dimensional characterization of its dimensions is essential for surgical and orthodontic planning. Two-dimensional radiographs underestimate bucco-lingual bone loss and cannot reliably depict the true architecture of the defect, which has driven a shift toward CBCT as the imaging modality of choice for cleft assessment. Comparative work evaluating different CBCT-based methods of quantifying alveolar cleft defects has confirmed that three-dimensional volumetric assessment is more accurate than any single-plane method [6], a conclusion echoed by a study comparing computer-simulation and water-displacement approaches to preoperative cleft-volume measurement on CBCT [7].

Cleft laterality itself appears to influence graft outcome and residual defect volume: a three-dimensional volumetric study of bilateral alveolar clefts managed with staged, two-step bone grafting found that the first-grafted site achieved significantly greater bone fill (47%) than the second-grafted site (33%), and that cleft volume on the still-ungrafted contralateral side did not change materially after the first surgery—evidence that bilateral clefts behave as two at least partly independent defects rather than a single symmetric entity, with direct relevance to how cleft laterality should be analyzed as a variable [8]. Corrected sagittal reconstruction, reoriented perpendicular to the dental arch rather than to the true anatomical plane, has become a standard adjunct to the axial view in these protocols because it allows unobstructed visualization of the cleft margins along the long axis of the adjacent roots [9].

The maxillary lateral incisor is the tooth most frequently affected by developmental disturbance in patients with cleft lip and palate, because its dental bud lies directly at or near the cleft margin. Using the Tooth Agenesis Code, López-Giménez et al. [10] reported agenesis prevalence of approximately 51% in both unilateral and bilateral cleft lip and palate in a Spanish cohort, with the lateral incisor the tooth most frequently missing, particularly within the cleft quadrant. Bartzela et al. [11] similarly found agenesis in nearly half of subjects in a Dutch cohort of complete unilateral cleft lip and palate patients, with the cleft-side lateral incisor absent in roughly two of five patients. A broader review of dental anomalies in cleft populations corroborated missing maxillary lateral incisors as the single most consistent finding across cleft severities and ethnicities, alongside supernumerary teeth, microdontia, and taurodontism [12].

Root resorption of a present lateral incisor may occur when it is positioned close to a displaced or erupting canine—a well-described phenomenon in the general orthodontic literature, where Alqerban et al. [13] synthesized the risk factors for lateral incisor root resorption caused by an adjacent impacted canine, including canine angulation, degree of radiographic overlap, and buccopalatal position. A large, more recent CBCT-based retrospective study of 305 patients with unilateral maxillary impacted canines confirmed that a thinner labial alveolar plate and a more labially inclined lateral incisor root are independently associated with a greater degree of resorption, and that resorption prevalence differs by the canine’s buccopalatal position [14]. In the cleft population specifically, the coexistence of a bony defect, a frequently rotated or displaced lateral incisor, and an atypical canine eruption path creates a distinct anatomical context in which this resorptive process has not been as systematically documented—a gap the present study addresses by classifying lateral incisor status as present, absent, or root-resorbed.

The maxillary canine in cleft patients follows an eruption pathway frequently altered by the adjacent bony defect, the presence or absence of the lateral incisor, and the timing of secondary alveolar bone grafting relative to root development. Oberoi et al. [9] found no significant association between congenital absence of the lateral incisor and canine root completion or eruption failure in a three-dimensional evaluation of the canine eruption pathway in bone-grafted alveolar clefts, whereas other investigators have reported an association between lateral incisor agenesis and an increased rate of canine impaction, albeit with low certainty of evidence. A systematic review by Grisar et al. [15] found that the initial three-dimensional position of impacted maxillary canines—angulation, vertical displacement, and mesial sector position—predicted treatment outcome across nearly 1600 impacted canines more strongly than eruption status alone. Importantly for interpreting eruption status in a mixed-dentition or early-adolescent cleft sample, an unerupted canine at the time of imaging does not necessarily indicate pathological impaction: physiological eruption of the maxillary canine typically occurs between 7 and 11 years of age, and a canine still descending along a normal path in a young patient should be classified as developing rather than impacted [5]. This distinction supports framing canine status in the present study as a descriptive, prevalence-based variable—erupted or unerupted—rather than a clinical diagnosis of impaction.

The nasopalatine (incisive) canal is a midline structure connecting the floor of the nasal cavity to the anterior hard palate, posterior to the maxillary central incisors. In non-cleft populations, CBCT morphometric studies describe substantial variation in canal shape, length, and diameter, with males generally exhibiting longer, wider canals than females [16], and morphologic canal change has been documented in relation to alveolar bone resorption and implant planning [17]. A study directly comparing the nasopalatine canal in patients with and without cleft palate, using CBCT exams evaluated in coronal, sagittal, and axial reformats, found funnel-, banana-, and needle-shaped canals significantly more frequent in cleft patients, together with a significantly larger nasopalatine foramen and canal diameter but a significantly shorter overall canal length compared with non-cleft controls [18]. A separate CBCT investigation that specifically evaluated cleft volume alongside adjacent anatomical structures found that the incisive foramen was not visible in 71% of bilateral cleft cases, significantly less often than in unilateral clefts, indicating that the cleft defect itself may directly involve or obliterate the canal’s oral opening in more severe presentations [19]. These findings collectively support the premise that the disrupted embryological fusion underlying the alveolar cleft also reshapes this adjacent midline canal, reinforcing the clinical relevance of characterizing the canal’s proximity to the cleft margin when planning bone grafting or implant-based rehabilitation.

Distortion of the nasal septum is one of the most consistent secondary deformities associated with cleft lip and palate, arising from both the primary embryological defect and the unopposed muscular pull exerted across the clefted lip and alveolus before surgical repair. An early study using direct visual scoring demonstrated that nasal septal deviation occurs significantly more frequently in individuals with cleft lip and palate than in non-cleft controls, with the cartilaginous nasal tip and anterior nasal spine both tending to deviate toward the non-cleft side [20]. Using CBCT, a widely cited otolaryngology study of nasal septal deviation in cleft palate and/or alveolus patients found that the degree of deviation correlated significantly with cleft severity [21], while a subsequent three-dimensional CBCT analysis quantified septal deviation angle and linear displacement at the anterior nasal spine, posterior nasal spine, and their midpoint, finding significantly greater deviation in unilateral cleft lip and palate patients than in skeletal Class III non-cleft controls [22]. The combined cleft-volume and adjacent-structures CBCT study noted above similarly found the nasal septum curved toward the cleft or graft side in 87% of unilateral cases, and additionally found the inferior turbinate significantly smaller on the cleft side than the non-cleft side [19].

Collectively, this literature establishes each variable relevant to the present study—alveolar cleft morphometrics, lateral incisor status, canine eruption pattern, nasopalatine canal morphology, and nasal septal deviation—as a recognized, independently investigated feature of the cleft lip and palate phenotype. However, few if any published studies have combined all of these parameters within a single cohort using a standardized CBCT protocol incorporating both axial and corrected coronal views, and fewer still have examined how lateral incisor status (present, missing, or root-resorbed) relates jointly to canine eruption pattern, cleft dimensions, and the position of two adjacent midline structures—the nasopalatine canal and the nasal septum—within the same patients. This integrated, prevalence-and-correlation approach—rather than a further single-structure analysis—is the specific contribution of the present study.

This scope also follows from the imaging modality: CBCT captures mineralized, bony structures well, but it does not reliably show the soft-tissue lip. CBCT-based bone-assessment protocols in cleft patients have therefore focused specifically on the alveolar and palatal skeleton [23,24]. For that reason, we scoped the present study to cleft palate—the bony component CBCT actually visualizes—rather than to cleft lip and palate as a combined soft- and hard-tissue diagnosis.

### 1.1. Objectives

The general objective of this study was to evaluate, using cone beam computed tomography, the Dento-alveolar and anatomical characteristics of patients with cleft palate and their statistical relationship to cleft laterality. The specific objectives were as follows:

1. To determine whether maxillary canine eruption status (erupted vs. unerupted) is statistically associated with lateral incisor status (present vs. absent) in patients with cleft palate, tested using Fisher’s exact test.
2. To measure the two-dimensional alveolar cleft width—the mesio-distal width on the axial plane and the bucco palatal depth on the corrected coronal plane—and to compare cleft width between unilateral and bilateral cleft cases.
3. To determine the prevalence of nasopalatine canal position and nasal septal deviation, and to test their statistical correlation with cleft laterality (unilateral right, unilateral left, or bilateral).

### 1.2. Hypotheses

Null Hypothesis (H0): Maxillary canine eruption status is not significantly associated with lateral incisor status; alveolar cleft width does not differ significantly between unilateral and bilateral cleft cases; and nasopalatine canal position and nasal septal deviation are not significantly correlated with cleft laterality.

Alternative Hypothesis (H1): Maxillary canine eruption status is significantly associated with lateral incisor status, such that an absent lateral incisor is associated with a different rate of canine non-eruption; alveolar cleft width differs significantly between unilateral and bilateral cleft cases; and nasopalatine canal position and nasal septal deviation are significantly correlated with cleft laterality.

## 2. Materials and Methods

### 2.1. Study Design and Setting

This retrospective cross-sectional study was conducted using CBCT examinations archived in the Oral Radiology Department, King Abdulaziz University Dental Hospital (KAUDH), Jeddah, Saudi Arabia. This report follows the STROBE (Strengthening the Reporting of Observational Studies in Epidemiology) guidelines for cross-sectional studies; the completed STROBE checklist is provided as a supplementary file, File S1.

### 2.2. Case Selection

Cases were identified through a retrospective search of the Oral Radiology Department archive for patients with cleft palate who had undergone CBCT imaging as part of routine multidisciplinary cleft care between August 2024 and June 2026.

To be included, a case needed a clinical diagnosis of cleft lip and/or palate, an archived CBCT examination acquired for routine multidisciplinary cleft care, and image quality and field of view sufficient to visualize the alveolar cleft together with the nasopalatine canal and nasal septum. Three situations led to exclusion: an alveolar cleft that had already been surgically closed before the CBCT was taken, a suspected cleft that turned out on review to be an isolated midline diastema, and any case where clinical and radiographic review did not confirm cleft palate. Since CBCT scan detects only bone structures, we studied only cleft palate without any assessment of lip cleft.

Thirty-six CBCT examinations initially met the search criteria. Three examinations were excluded before analysis: one because the alveolar cleft had already been surgically closed before CBCT acquisition, one because the finding initially interpreted as a cleft represented an isolated midline diastema, and one because review of the clinical and radiographic records confirmed that the patient did not have a cleft palate. The remaining 33 examinations constituted the eligible analytic cohort (Figure 1). We did not perform an a priori sample-size calculation. The study simply included every eligible examination available in the archive over the study period, which fits the exploratory, retrospective nature of this analysis. Section 4 discusses the resulting sample size and the limited power this leaves for the smaller subgroups.

The following variables were available for the eligible cohort: sex, age at imaging, cleft laterality, maxillary lateral incisor status, maxillary canine status, nasopalatine canal position, and nasal septal deviation.

Because tooth development may be incomplete in younger patients, particular caution was applied to the interpretation of root resorption and eruption status. Six patients (18.2%) were 7–8 years of age at the time of imaging. These patients were retained in the primary analyses, but findings dependent on root development or physiological eruption timing were interpreted with consideration of developmental stage.

When more than one eligible CBCT examination was available for the same patient, the examination selected for analysis was the earliest eligible pretreatment examination that provided adequate visualization of the structures required for the study. When multiple examinations were available around the same treatment milestone, the examination with more complete dental development and adequate image quality was preferentially selected.

### 2.3. CBCT Acquisition and Image Evaluation

CBCT examinations had been acquired using equipment available in the Oral Radiology Department during the study period. Most examinations were obtained using a KaVo 3D eXam^®^ CBCT unit (KaVo Dental GmbH, Biberach, Germany), while a subset had been acquired using an i-CAT^®^ unit (Imaging Sciences International/KaVo Kerr, Hatfield, PA, USA).

For examinations acquired using the KaVo 3D eXam unit, the standardized exposure parameters were 90 kV, 6.3 mA, and an exposure time of 8.7 s, with a dose–area product of 509 mGy/cm^2^. Because the study used a retrospective clinical archive, field of view was not uniform across examinations. However, all included examinations encompassed the bilateral maxilla, allowing assessment of both the cleft-affected and contralateral regions. The majority of examinations used an isotropic voxel size of 0.3 mm.

CBCT datasets were reviewed using OnDemand3D imaging software (Cybermed Inc., Seoul, South Korea). Images were evaluated using multiplanar reconstruction (MPR) in axial, sagittal, and coronal planes (Figure 2). Panoramic reconstructions were also reviewed when useful for assessment of tooth presence, eruption status, and overall dental anatomy.

Patient age and sex were obtained from the clinical imaging records and cross-checked against the archived patient information.

### 2.4. Measurement Protocol and Reference Planes

A standardized image-orientation protocol was used for all linear measurements.

For sagittal assessment, the horizontal reference line was oriented parallel to the floor of the hard palate. For axial and coronal assessment, the anatomical midline was defined using the midpoint of the nasal septum. The anatomical midline was used rather than the dental midline because cleft-related displacement of the anterior dentition may alter the position of the dental midline. Because this same septal reference was also used to classify nasal septal position (Section 2.5), the nasopalatine canal—an independent structure—was assessed against this midline without circularity, but nasal septal position could not be assessed against a fully independent skeletal landmark in this retrospective dataset; this is addressed explicitly as a limitation (Section 4).

Two linear cleft measurements were evaluated:

1. **Axial mesiodistal cleft width:** measured on the axial CBCT reconstruction at the coronal third of the alveolar process, approximately 1 mm apical to the alveolar crest. The measurement represented the mesiodistal distance across the alveolar cleft between the central incisor and the adjacent lateral incisor or canine bordering the defect (Figure 3).

2. **Corrected-coronal buccopalatal cleft depth:** measured on a corrected coronal reconstruction at the mid-palatal level. The corrected coronal plane was oriented perpendicular to the dental arch and positioned to pass through the relevant adjacent tooth structures. The measurement was obtained distal to the root of the lateral incisor when the lateral incisor was present or relative to the canine when the lateral incisor was absent. The corrected coronal reconstruction was used to obtain a plane perpendicular to the relevant dental anatomy and to minimize measurement distortion caused by the three-dimensional orientation of the cleft.

In bilateral cleft cases in which the two cleft spaces formed a continuous defect, the defect was evaluated relative to the anatomical midline and the measurements were recorded separately for the right and left components where the anatomy allowed side-specific assessment.

Axial cleft-width measurements were available for 45 cleft-side observations. Corrected-coronal buccopalatal depth measurements were available for 44 cleft-side observations. A “cleft-side observation” refers to one measurable cleft margin; unilateral patients contributed one such observation and bilateral patients could contribute up to two, which is why these counts exceed the 33-patient cohort.

### 2.5. Variables and Classification

The following variables were recorded for each patient:

- sex;
- age at CBCT imaging;
- cleft laterality (unilateral right, unilateral left, or bilateral);
- maxillary lateral incisor status for teeth #12 and #22;
- maxillary canine status for teeth #13 and #23;
- nasopalatine canal position; and
- nasal septal deviation.

**Maxillary lateral incisor status**

Each maxillary lateral incisor was classified as follows:

- present without apical root resorption;
- present with apical root resorption; or
- absent at the time of imaging.

For statistical analyses, lateral incisor status was further dichotomized as **present** or **absent**. Among present lateral incisors, root resorption was analyzed as a separate binary variable.

The term **absent** refers to failure to identify the permanent lateral incisor at the time of CBCT examination and does not necessarily establish congenital agenesis, particularly in younger patients. In cases in which a retained primary lateral incisor could not be confidently distinguished from a developing permanent successor, the permanent lateral incisor was recorded as absent for the radiographic time point rather than being interpreted as confirmed congenital agenesis (Figure 4).

**Maxillary canine status**

Each maxillary canine was classified as either **erupted** or **unerupted**.

The unerupted category included canines that remained intraosseous at the time of CBCT examination. This classification was deliberately used instead of “impacted” because a single CBCT examination cannot reliably distinguish pathological impaction from normal developmental non-eruption, particularly in younger patients.

**Nasopalatine canal position**

The nasopalatine canal was classified according to its position relative to the anatomical midline as follows:

- deviated toward the right;
- centered/midline; or
- deviated toward the left.

When the canal could not be confidently localized, the anatomical midline was used as the reference and the canal was classified as centered rather than assigning an uncertain directional deviation.

**Nasal septal deviation**

Nasal septal position was classified as follows:

- deviated toward the right;
- deviated toward the left; or
- no appreciable deviation.

Because the anatomical midline above (Section 2.4) was itself derived from the nasal septum, this variable describes the septum’s course relative to the study’s own reference rather than displacement validated against a wholly independent skeletal axis; this is discussed as a limitation in Section 4.

### 2.6. Examiner Reliability and Data Quality Control

All image assessments were performed by a single oral and maxillofacial radiologist (H.S.).

To assess intra-examiner reliability, a subset of examinations was re-evaluated after an interval sufficient to minimize recall of the initial measurements. The repeated assessments included axial cleft width and corrected-coronal buccopalatal depth measurements. The number of repeated cases ranged from 10 to 13 for each measurement category.

Reliability was assessed using a two-way mixed-effects intraclass correlation coefficient for absolute agreement and single measurements [ICC(A,1)]. Mean signed differences between the first and second measurements and 95% limits of agreement were also calculated.

All measurements were subsequently checked against the original CBCT examinations and source data before statistical analysis. Any obvious data-entry discrepancies were resolved by returning to the original CBCT dataset and recording the verified measurement.

For nasopalatine canal position and nasal septal deviation—the two categorical variables underlying the principal significant findings of this study—intra-examiner reliability was additionally assessed via a blinded re-read of all 33 cases, with agreement quantified using Cohen’s kappa: κ = 0.86 for canal position and κ = 0.88 for septal deviation (both almost-perfect agreement; see Section 3.3.1). Repeat categorical readings were not available for canine or lateral incisor status in this retrospective dataset.

### 2.7. Statistical Analysis

Canine and lateral incisor status belong to a specific tooth and side, so we analyzed these two variables at the tooth-side level. Cleft laterality, cleft width, nasopalatine canal position, and nasal septal deviation, by contrast, are properties of the patient as a whole rather than of one tooth, so we analyzed these at the patient level. We kept this distinction throughout the analyses below. Descriptive statistics were calculated for all variables. Categorical variables were summarized using frequencies and percentages. Continuous variables were summarized using the mean, standard deviation, median, and range.

For Objective 1, the association between ipsilateral lateral incisor status (present vs. absent) and canine eruption status (erupted vs. unerupted) was assessed separately for the right side (#12/#13) and left side (#22/#23) using Fisher’s exact test. Because right and left tooth-side observations from the same patient are not statistically independent, pooled bilateral tooth-side data were presented descriptively but were not subjected to an additional inferential Fisher’s exact test.

At the patient level, chi-square tests were used to assess the relationship between cleft laterality and the presence of at least one absent lateral incisor and between cleft laterality and the presence of at least one unerupted canine. Fisher’s exact test was used to evaluate the relationship between sex and the presence of at least one absent lateral incisor.

Age was compared between patients with and without at least one absent lateral incisor, with and without root resorption, and with at least one unerupted canine versus both canines erupted using the Mann–Whitney U test.

The relationship between the specific cleft-affected side and tooth-level findings was assessed descriptively and, where appropriate, using Fisher’s exact test because bilateral cases contribute observations from both sides and therefore do not represent independent patient-level observations.

For Objective 2, axial cleft width and corrected-coronal buccopalatal depth were summarized by side. For comparison by cleft laterality, axial width was averaged across available cleft sides for each patient, and the resulting patient-level values were compared among unilateral-left, unilateral-right, and bilateral cleft groups using the Kruskal–Wallis test. A simplified unilateral-versus-bilateral comparison was additionally performed using the Mann–Whitney U test. The relationship between patient age and mean axial cleft width was assessed using Spearman’s rank correlation coefficient.

For Objective 3, the associations between nasopalatine canal position and cleft laterality and between nasal septal deviation and cleft laterality were assessed using the Fisher–Freeman–Halton exact test because of the sparse cell counts in the 3 × 3 contingency tables. Pearson chi-square statistics were also calculated as reference statistics. Among unilateral cases, the direction of nasopalatine canal position and nasal septal deviation relative to the cleft side was summarized descriptively as same-side or opposite-side deviation.

A two-sided *p*-value < 0.05 was considered statistically significant. Because several analyses were exploratory and the study was based on a relatively small retrospective cohort, *p*-values were interpreted in the context of effect direction, sample size, and measurement availability rather than as definitive evidence of absence of association.

Statistical analyses were performed using Python version 3.11 with the pandas and SciPy libraries.

During preparation of this manuscript, the author used an AI-based writing and analysis assistant Claude Sonnet 5 (Anthropic) for literature search support, statistical cross-checking, and language editing assistance. The authors have reviewed and edited the output and take full responsibility for the content of this publication.

## 3. Results

### 3.1. Sample Characteristics

Of the 36 CBCT examinations initially identified, 3 were excluded (Section 2.2 details the inclusion/exclusion criteria and reasons); the final analytic cohort comprised 33 patients (Figure 1).

The cohort included 17 females (51.5%) and 16 males (48.5%). The mean age at imaging was 12.5 ± 4.1 years, with a median of 12.0 years and a range of 7–23 years. Six patients (18.2%) were 7–8 years old at imaging.

Unilateral left-sided clefts were the most common presentation (*n* = 14, 42.4%), followed by bilateral clefts (*n* = 12, 36.4%) and unilateral right-sided clefts (*n* = 7, 21.2%) (Table 1).

### 3.2. Objective 1: Maxillary Lateral Incisor and Canine Status

For tooth #12, 18 of 33 patients (54.5%) had a lateral incisor without apical root resorption, 2 (6.1%) had apical root resorption, and 13 (39.4%) had no identifiable permanent lateral incisor at the time of imaging.

For tooth #22, 15 patients (45.5%) had a lateral incisor without apical root resorption, 3 (9.1%) had apical root resorption, and 15 (45.5%) had no identifiable permanent lateral incisor.

When both sides were considered descriptively, 28 of 66 tooth-side observations (42.4%) were classified as absent and 38 (57.6%) as present (Table 2).

Maxillary canine status was classified as erupted or unerupted. For tooth #13, 20 patients (60.6%) had an unerupted canine and 13 (39.4%) had an erupted canine. For tooth #23, 19 patients (57.6%) had an unerupted canine and 14 (42.4%) had an erupted canine. No congenitally absent maxillary canine was identified in the cohort (Table 3).

On the right side, an unerupted #13 was present in 8 of 13 cases (61.5%) in which #12 was absent, compared with 12 of 20 cases (60.0%) in which #12 was present (Fisher’s exact *p* = 1.000).

On the left side, an unerupted #23 was present in 8 of 15 cases (53.3%) in which #22 was absent, compared with 11 of 18 cases (61.1%) in which #22 was present (Fisher’s exact *p* = 0.733).

Descriptively, when both sides were pooled, 16 of 28 tooth-side observations with an absent lateral incisor (57.1%) had an unerupted canine, compared with 23 of 38 observations with a present lateral incisor (60.5%). Because the two sides from an individual patient are not independent observations, no inferential *p*-value was assigned to this pooled analysis (Table 4).

Among the 38 tooth-side observations in which the lateral incisor was present, apical root resorption was identified in 5 (13.2%). Resorption was observed in 2 of 20 present right lateral incisors (10.0%) and 3 of 18 present left lateral incisors (16.7%) (Table 5).

#### 3.2.1. Patient-Level Prevalence of Missing Lateral Incisors

At the patient level, 18 patients (54.5%) had both lateral incisors present, 13 (39.4%) had both lateral incisors absent, and 2 (6.1%) had one absent and one present lateral incisor. Thus, at least one lateral incisor was absent in 15 of 33 patients (45.5%) (Table 6).

Mean age did not differ significantly between patients with at least one absent lateral incisor and those with both lateral incisors present (12.1 ± 3.7 vs. 12.8 ± 4.5 years; Mann–Whitney U = 124.0, *p* = 0.703).

The proportion of patients with at least one absent lateral incisor was 9 of 17 females (52.9%) and 6 of 16 males (37.5%); this difference was not statistically significant (Fisher’s exact *p* = 0.491) (Table 7).

#### 3.2.2. Relationship of Cleft Laterality and Age with Dentoalveolar Findings

At the tooth-side level, lateral incisor absence was observed in 20 of 45 cleft-affected sides (44.4%) compared with 8 of 21 non-cleft sides (38.1%). This difference was not statistically significant (χ^2^ = 0.048, *p* = 0.827).

Among present lateral incisors, apical root resorption was observed in 5 of 25 cleft-affected teeth (20.0%) compared with none of 13 present lateral incisors on non-cleft sides. Fisher’s exact test did not demonstrate a statistically significant association (*p* = 0.144).

An unerupted canine was identified on 28 of 45 cleft-affected sides (62.2%) compared with 11 of 21 non-cleft sides (52.4%); this difference was not statistically significant (χ^2^ = 0.239, *p* = 0.625).

At the patient level, cleft laterality was not significantly associated with the presence of at least one absent lateral incisor (χ^2^ = 2.510, df = 2, *p* = 0.285) or with the presence of at least one unerupted canine (χ^2^ = 1.736, df = 2, *p* = 0.420).

Patient age was significantly associated with canine eruption status. Patients with at least one unerupted canine were younger than patients with both canines erupted (10.8 ± 3.3 vs. 15.1 ± 4.1 years; Mann–Whitney U = 53.5, *p* = 0.005).

Age was not significantly associated with root resorption. Patients with any root resorption had a mean age of 15.7 ± 7.5 years (*n* = 3), compared with 12.2 ± 3.7 years (*n* = 30) among those without resorption (Mann–Whitney U = 58.5, *p* = 0.414). This comparison was limited by the small number of patients with root resorption (Table 8).

#### 3.2.3. Canine Status by Age and Sex

At the patient level, the proportion of patients with at least one unerupted canine was highest in the younger age groups. An unerupted canine was present in 9 of 10 patients aged 7–9 years (90.0%), 6 of 8 patients aged 10–12 years (75.0%), 2 of 7 patients aged 13–15 years (28.6%), and 3 of 8 patients aged 16 years or older (37.5%) (Table 9).

Among females, 10 of 17 patients (58.8%) had at least one unerupted canine, compared with 10 of 16 males (62.5%). This difference was not statistically significant (Fisher’s exact *p* = 1.000) (Table 10).

### 3.3. Objective 2: Alveolar Cleft Dimensions

Axial mesiodistal cleft-width measurements were available for 19 right-sided and 26 left-sided cleft margins, giving 45 side-level observations. Corrected-coronal buccopalatal depth measurements were available for 19 right-sided and 25 left-sided cleft margins, giving 44 observations.

The mean axial cleft width was 6.29 ± 2.72 mm (median, 5.95 mm; range, 2.20–12.50 mm; *n* = 45). The mean corrected-coronal buccopalatal depth was 8.04 ± 5.30 mm (median, 6.30 mm; range, 1.30–27.00 mm; *n* = 44) (Table 11).

For patient-level comparison by cleft laterality, axial cleft width was averaged across available cleft sides for each patient. Mean axial width was 5.51 ± 3.18 mm among patients with unilateral left-sided clefts (*n* = 14), 5.59 ± 1.32 mm among patients with unilateral right-sided clefts (*n* = 7), and 7.15 ± 2.65 mm among patients with bilateral clefts (*n* = 12).

The three-group comparison did not demonstrate a statistically significant difference (Kruskal–Wallis H = 3.79, df = 2, *p* = 0.150).

In the simplified comparison of unilateral versus bilateral clefts, mean axial width was 5.54 ± 2.67 mm for unilateral clefts and 7.15 ± 2.65 mm for bilateral clefts (Mann–Whitney U = 174.5, *p* = 0.072).

Mean axial cleft width was not significantly correlated with patient age (Spearman’s ρ = 0.158, *n* = 33, *p* = 0.378) (Table 12).

#### 3.3.1. Intra-Examiner Reliability

Intra-examiner reliability was high for both axial cleft-width and corrected-coronal depth measurements.

For right-sided axial width measurements, the ICC was 0.942 (95% CI, 0.82–0.98; *n* = 13). For right-sided corrected-coronal depth, the ICC was 0.994 (95% CI, 0.98–1.00; *n* = 12).

For left-sided axial width, the ICC was 0.970 (95% CI, 0.89–0.99; *n* = 11), while the ICC for left-sided corrected-coronal depth was 0.976 (95% CI, 0.91–0.99; *n* = 10).

Across the paired measurements, the overall ICC was 0.974 (95% CI, 0.95–0.99). The mean signed difference between repeated measurements was 0.11 mm, with 95% limits of agreement from −0.84 to 1.06 mm.

Categorical reliability was assessed via a blinded re-read of all 33 cases, performed after an interval sufficient to minimize recall. Cohen’s kappa was 0.86 for nasopalatine canal position and 0.88 for nasal septal deviation, both indicating almost-perfect agreement (Landis and Koch benchmarks); paired repeat readings were not available for canine or lateral incisor status in this dataset.

These results indicate high intra-examiner agreement for the linear cleft measurements.

#### 3.3.2. Cleft Width According to Sex and Adjacent Anatomical Findings

Mean axial cleft width was 5.94 ± 3.18 mm among females and 6.29 ± 2.28 mm among males (Mann–Whitney U = 114.5, *p* = 0.449).

Mean corrected-coronal depth was 7.72 ± 5.98 mm among females and 7.56 ± 3.27 mm among males (Mann–Whitney U = 116.5, *p* = 0.494).

Axial cleft width differed according to nasopalatine canal position (Kruskal–Wallis H = 7.44, *p* = 0.024). Mean axial width was 7.82 ± 2.79 mm when the canal was centered, 4.87 ± 2.37 mm when the canal deviated to the right, and 5.93 ± 2.28 mm when it deviated to the left.

Corrected-coronal depth did not differ significantly according to nasopalatine canal position (H = 2.39, *p* = 0.302).

Neither axial cleft width nor corrected-coronal depth differed significantly according to nasal septal deviation (H = 0.13, *p* = 0.721 and H = 0.05, *p* = 0.827, respectively) (Table 13).

### 3.4. Objective 3: Nasopalatine Canal Position and Nasal Septal Deviation

Overall, the nasopalatine canal was positioned toward the right in 14 patients (42.4%), centered in 11 patients (33.3%), and toward the left in 8 patients (24.2%). Of the 11 centrally classified canals, 9 were centered on both the primary and blinded repeat reading (confirmed midline) and 2 were reclassified as laterally deviated on repeat reading (indeterminate rather than truly centered); all 11 were retained within the centered category for the primary analysis (Section 4).

The nasal septum deviated toward the left in 21 patients (63.6%), toward the right in 11 patients (33.3%), and showed no appreciable deviation in 1 patient (3.0%) (Table 14).

Nasopalatine canal position was significantly associated with cleft laterality. The Pearson chi-square statistic was χ^2^ = 15.94 with 4 degrees of freedom (*p* = 0.003), while the Fisher–Freeman–Halton exact test yielded *p* = 0.002 (Table 15).

Nasal septal deviation was also significantly associated with cleft laterality. The Pearson chi-square statistic was χ^2^ = 15.00 with 4 degrees of freedom (*p* = 0.005), while the Fisher–Freeman–Halton exact test yielded *p* = 0.001 (Table 16).

Among the 21 unilateral cases, 16 had a clearly directional nasopalatine canal position after excluding centrally positioned canals. The canal was positioned on the side opposite the cleft in 14 of these 16 cases (87.5%) and on the same side in 2 cases (12.5%).

Nasal septal deviation was toward the same side as the cleft in 19 of the 21 unilateral cases (90.5%) and toward the opposite side in 2 cases (9.5%).

### 3.5. Sensitivity Analysis by Age

Because six patients were 7–8 years old, a sensitivity analysis was performed after excluding these younger patients. The resulting subgroup comprised 27 patients aged 9–23 years.

The association between lateral incisor status and canine eruption status remained non-significant in the older subgroup. The age difference between patients with an unerupted canine and those with both canines erupted remained significant, with mean ages of approximately 11.9 and 15.7 years, respectively (Mann–Whitney U = 37.0, *p* = 0.010).

Root resorption was observed in 3 of 31 present lateral incisors (9.7%) in the older subgroup, compared with 5 of 38 (13.2%) in the full dataset.

The associations between cleft laterality and nasopalatine canal position and between cleft laterality and nasal septal deviation also remained statistically significant in the older subgroup (exact *p* = 0.005 and *p* = 0.004, respectively).

Overall, exclusion of the youngest patients did not materially alter the direction of the principal findings. Figure 5 summarizes these primary comparisons by cleft laterality: the two significant, reciprocal findings for nasopalatine canal position (A) and nasal septal deviation (B), the significant association between age and canine eruption status (C), and the non-significant cleft-width comparison (D) shown for context.

## 4. Discussion

This retrospective CBCT analysis of 33 patients with cleft palate examined the relationship between maxillary canine and lateral incisor status, the two-dimensional dimensions of the alveolar cleft, and the position of two adjacent midline structures—the nasopalatine canal and the nasal septum—in relation to cleft laterality, age, and sex. The distribution of cleft laterality in this cohort (left-sided in 42.4%, bilateral in 36.4%, right-sided in 21.2%) is broadly consistent with the wider epidemiological literature on cleft palate, in which left-sided unilateral involvement is consistently reported as the most common presentation, even as birth prevalence and phenotypic distribution vary by population [1,2,3,4].

The prevalence of lateral incisor absence observed here—45.5% of patients missing at least one lateral incisor, and 39.4% missing both—sits within the range reported in larger cleft cohorts using comparable tooth-agenesis classification systems. Using the Tooth Agenesis Code, López-Giménez et al. [10] reported agenesis in approximately 51% of unilateral and bilateral cleft lip and palate patients, with the lateral incisor the tooth most frequently affected, while Bartzela et al. [11] found agenesis in nearly half of a Dutch unilateral cleft cohort, with the cleft-side lateral incisor absent in roughly two of five patients, and Haque and Alam [12] identified missing maxillary lateral incisors as the single most consistent dental anomaly across cleft severities and populations. The present findings therefore corroborate this literature rather than diverging from it, reinforcing that lateral incisor absence—whether reflecting true congenital agenesis or non-visualization at the time of imaging (Section 2.5)—should be anticipated in close to half of cleft palate patients regardless of the specific population studied. Canine status in this cohort was weighted toward the unerupted category (60.6% right, 54.5% left; one left canine, 3.0%, was congenitally absent), consistent with the broader recognition that eruption of the maxillary canine adjacent to an alveolar cleft is frequently disrupted [25]. Critically, however, canine status was not significantly associated with lateral incisor status in this sample, whether tested per side (right side *p* = 1.000; left side *p* = 0.733); because the two sides of an individual patient are not independent observations, no inferential test was performed on the pooled tooth-side data (Section 3.2). This null finding is concordant with Oberoi et al. [9], who, using three-dimensional CBCT assessment of the canine eruption pathway in bone-grafted alveolar clefts, likewise found no significant association between congenital absence of the lateral incisor and canine eruption failure; together, the two studies suggest that canine eruption disturbance in cleft patients may be driven predominantly by the alveolar bony defect itself and the timing of bone grafting, rather than by the presence or absence of the adjacent lateral incisor. This contrasts with the general orthodontic literature outside the cleft population, where the initial three-dimensional position of an impacted canine—angulation, vertical displacement, mesial sector position—has been shown to be the dominant prognostic factor for eruption success [15], again pointing to canine position itself, rather than neighboring tooth status, as the more clinically actionable variable.

Apical root resorption was identified in 13.2% of lateral incisors classified as present, a figure lower than resorption rates sometimes reported in general (non-cleft) populations with impacted canines, where thinner labial alveolar bone and more labially inclined lateral incisor roots have been shown to substantially increase resorption risk [14], and where canine angulation and degree of radiographic overlap are established risk factors [13]. Because the present study did not measure alveolar bone thickness or root inclination, a direct mechanistic comparison with these bone-thickness-driven models is not possible; the comparatively lower resorption prevalence observed here may in part reflect the relatively young age of several cases (18.2% imaged at 7–8 years, before root maturity is reliably complete), which would tend to under-detect resorption that develops later as the canine continues its eruptive path. Consistent with this developmental interpretation, patient age was not significantly associated with lateral incisor status (*p* = 0.703) or root resorption (*p* = 0.414), but was significantly associated with canine status (*p* = 0.005): younger patients were substantially more likely to present with an unerupted canine (mean age 10.8 years) than an erupted one (mean age 15.1 years), while sex showed no such association (*p* = 1.000). This age effect is best explained by normal physiological eruption timing—the maxillary canine typically erupts between 7 and 11 years of age [5]—rather than a cleft-specific pathological process, and underscores the importance of age-stratified interpretation when canine eruption status is used as an outcome measure in cleft research. Neither cleft laterality nor the specific side affected by the cleft was significantly associated with lateral incisor status, root resorption, or canine status in this sample (all *p* > 0.14), suggesting that at the sample size available here, these dentoalveolar anomalies behave more as a generalized feature of the cleft phenotype than as a strictly lateralized, mechanically localized effect.

Turning from the dentition to the dimensions of the cleft itself, mean axial cleft width in this cohort (6.29 ± 2.72 mm pooled across sides) is broadly comparable in order of magnitude to alveolar cleft dimensions reported in other CBCT-based cohorts using three-dimensional or multi-planar measurement protocols, which have consistently favored volumetric or multi-plane assessment over single linear measurements for capturing the true extent of the defect [6,7]. Cleft width did not differ significantly across left-sided, right-sided, and bilateral cleft groups (Kruskal–Wallis *p* = 0.150), nor between unilateral and bilateral cleft considered as a simplified two-group comparison (*p* = 0.072), although bilateral clefts were numerically the widest of the three groups (7.15 mm vs. 5.51–5.59 mm for left and right unilateral clefts, respectively); nor was cleft width significantly associated with age (Spearman’s rho = 0.158, *p* = 0.378) or sex (*p* = 0.449). The non-significant bilateral-wider trend is directionally consistent with volumetric evidence from Regnstrand, Bousiou et al. (2025) [8], who used three-dimensional CBCT to show that bilateral alveolar clefts behave as two only partly independent defects with unequal bone-graft fill between the first- and second-grafted sides (47% vs. 33%), implying that bilateral clefts are architecturally more complex than unilateral clefts even when overall linear width is similar; the absence of statistical significance in the present comparison is most plausibly attributable to sample size (12 bilateral and 21 unilateral patients) rather than a true absence of an underlying difference, and should be revisited once cleft width data collection is complete for the full cohort. One further, unexpected association emerged when cleft width was tested against nasopalatine canal position: axial width differed significantly across the three canal-position groups (Kruskal–Wallis H = 7.44, *p* = 0.024), with a centrally positioned canal associated with the widest clefts. This should be interpreted cautiously, however, since a centrally positioned canal was itself more common in bilateral cleft cases (50.0%) than in unilateral cleft (35.7% left-sided, 0.0% right-sided), and bilateral clefts were independently the widest laterality group; the width–canal-position association therefore most plausibly reflects this shared relationship with bilaterality rather than an independent mechanistic link between cleft size and canal position. No comparable prior study in the reviewed literature appears to have directly tested the correlation between cleft width and nasopalatine canal or nasal septal position, so this association—and its likely confounding by laterality—is presented here as a preliminary, hypothesis-generating observation rather than an established finding, and coronal depth showed no corresponding association with either structure (both *p* > 0.30).

The strongest and most consistent findings in this study concerned the two midline structures adjacent to the cleft. Both the nasopalatine canal and the nasal septum showed a statistically significant relationship with cleft laterality (exact Fisher–Freeman–Halton *p* = 0.002 and *p* = 0.001, respectively), and the two structures deviated in reciprocal directions relative to the cleft side: among unilateral cases, the nasopalatine canal deviated away from the cleft side in 87.5% of assessable cases, while the nasal septum deviated toward the cleft side in 90.5% of cases. The magnitude and direction of the septal finding align closely with Vicente, Wiedel et al. [19], who reported the nasal septum curved toward the cleft or graft side in 87% of unilateral cases using a comparable CBCT-based evaluation of cleft volume and adjacent anatomical structures—a striking convergence between two independent cohorts. Other CBCT-based investigations have similarly established that nasal septal deviation is significantly more pronounced in cleft lip and palate patients than in non-cleft or skeletal Class III controls [22], and that the degree of deviation correlates with cleft severity [21], lending further support to the present finding that septal position is systematically tied to the underlying cleft anatomy rather than incidental. It is worth noting that earlier work using direct visual scoring reported the cartilaginous nasal tip and anterior nasal spine deviating toward the non-cleft side [20]—apparently the opposite direction to the septal body deviation observed here and by Vicente, Wiedel et al. [19]. Rather than a genuine contradiction, this most likely reflects that the nasal tip, anterior nasal spine, and posterior/mid-septal body are anatomically distinct landmarks that can deviate along different, even opposing, trajectories within a single S- or C-shaped deformity, as is well recognized in cleft rhinoseptal anatomy; the present study measured septal position at the level of the nasal floor on axial and coronal CBCT, which is not directly equivalent to nasal tip or anterior nasal spine position on clinical or photographic assessment. The nasopalatine canal findings extend previous CBCT-based work demonstrating that canal morphology itself—not only its position—is altered in cleft patients, with funnel-, banana-, and needle-shaped canals, a larger foramen and canal diameter, and a shorter overall canal length all reported significantly more frequently in cleft than non-cleft controls [18]; the present study adds a laterality-specific dimension to this picture, showing that the canal’s position within the anterior maxilla is systematically displaced away from the cleft side in unilateral cases. Combined with the reciprocal septal finding, this pattern is consistent with the alveolar and palatal bony defect exerting an asymmetric influence on the entire anterior nasal floor complex, displacing the septum toward the defect while the bony canal is displaced away from it, rather than the two structures being affected independently. From a clinical standpoint, this reciprocal relationship may be of practical value in presurgical CBCT planning in at least three concrete ways. First, when planning the surgical approach and graft harvest trajectory for secondary alveolar bone grafting, awareness that the nasopalatine canal is statistically more likely to lie toward the non-cleft side in unilateral cases can help the surgeon anticipate its position on the preoperative scan and avoid inadvertent canal violation during graft-bed preparation, particularly in cases where the canal is difficult to trace directly, an application directly relevant to the surgical planning literature on secondary alveolar bone grafting [8,26]. Second, if future implant-based rehabilitation of the anterior maxilla is anticipated once the patient reaches skeletal maturity, the same laterality-dependent canal position could inform where implant osteotomies are more or less likely to encroach on the canal, complementing existing morphometric data on canal shape and diameter in cleft patients [18]. Third, the systematic septal deviation toward the cleft side is a reminder to orthodontists and rhinologists coordinating presurgical orthopedics or septorhinoplasty timing that nasal airway assessment should routinely accompany dento-alveolar planning in this population, since the two structures appear to be displaced by a shared mechanism rather than varying independently. These are proposed clinical applications rather than tested interventions, and each would need to be evaluated prospectively—for example, by comparing planned versus actual canal position on intraoperative navigation, or by correlating septal deviation with subjective nasal airway outcomes—before being adopted as formal planning guidance.

Beyond these procedural applications, the systematic and reciprocal relationship observed here between cleft laterality and the position of two independent midline structures carries a broader interpretive implication. If cleft lip and palate were purely a localized failure of alveolar and palatal fusion, there is no obvious mechanical reason why the nasopalatine canal and nasal septum—structures that lie outside the cleft margin itself—should shift in a laterality-dependent, reciprocal pattern. That they do so—consistently across this cohort and convergent with an independent cohort [19]—suggests cleft lip and palate disturbs the anterior nasomaxillary complex as a whole, not just the alveolar segment. This interpretation is reinforced by several recent CBCT studies reporting nasal septal, nasal complex, and maxillary sinus asymmetry in cleft cohorts [27,28,29,30], although, as in the present study, these structures have generally been examined individually rather than alongside the dentoalveolar cleft itself. Recent CBCT and digital-model evidence on the dentoalveolar side of this picture points the same way: agenesis patterns continue to be reported by cleft laterality [31], and canine-substitution approaches for the agenesis space remain an active area of study [32], reinforcing that both the dentoalveolar and midline findings in cleft lip and palate warrant continued, laterality-specific characterization. This does not make the alveolar cleft any less clinically important, but it is a reason for CBCT protocols in cleft care to routinely capture the nasopalatine canal and nasal septum alongside the cleft itself—both as a diagnostic habit, and as a further test of this model in larger cohorts.

### Study Limitations

This study has several limitations. First, the sample size was relatively small (*n* = 33), with particularly small subgroups for unilateral right-sided (*n* = 7) and bilateral (*n* = 12) clefts. Therefore, the study may have had limited power to detect modest associations, and non-significant findings should not be interpreted as evidence that no association exists. Several secondary analyses were exploratory, and no adjustment for multiple comparisons was performed.

Second, the retrospective single-center design limits generalizability. CBCT examinations were acquired for clinical purposes using non-uniform imaging protocols, including variation in field of view and CBCT unit, rather than under a standardized research protocol.

Third, although cleft measurements were largely complete, one anatomically eligible side could not be measured because of insufficient dental development to identify reliable measurement landmarks. In addition, one erroneous measurement recorded for a non-cleft side was identified during data verification and excluded from analysis. These issues were limited to individual measurements and did not alter the reported results.

Fourth, the cohort included patients aged 7–23 years, with six patients aged 7–8 years. Variation in dental developmental stage may affect interpretation of root resorption, lateral incisor development, and canine eruption. The sensitivity analysis excluding these younger patients showed that the principal findings, including the associations between cleft laterality and nasopalatine canal position and nasal septal deviation, remained statistically significant; however, the small sample limits the ability to exclude subtler age-related effects.

Fifth, all image assessments were performed by a single examiner. Intra-examiner reliability for the linear cleft measurements was excellent (pooled ICC(A,1) = 0.974; 95% CI, 0.95–0.99). For the two categorical variables underlying the principal significant findings of this study—nasopalatine canal position and nasal septal deviation—intra-examiner reliability was assessed via a blinded re-read of all 33 cases performed after an interval sufficient to minimize recall, with agreement quantified using Cohen’s kappa: κ = 0.86 for canal position and κ = 0.88 for septal deviation, both indicating almost-perfect agreement. Repeat categorical readings were not available for canine or lateral incisor status in this retrospective dataset, so their reliability could not be similarly quantified; this remains noted as a residual limitation.

Sixth, two aspects of midline-structure classification warrant caution. The anatomical midline used for both nasopalatine canal position and nasal septal deviation was defined from the midpoint of the nasal septum itself (Section 2.4); this is not circular for the canal, an independent structure, but it means septal position describes the septum’s course relative to this study’s own reference rather than displacement from a wholly independent skeletal axis. Separately, canal cases in which position could not be confidently lateralized were classified as centered together with cases in which the canal was genuinely at the midline (Section 2.5). Using agreement between the primary and blinded repeat reading (Section 2.6) as an operational criterion, 9 of the 11 canals classified as centered were centered on both readings (confirmed midline), while the remaining 2 were reclassified as laterally deviated on repeat reading (indeterminate rather than truly centered; Section 3.4). An independently defined skeletal landmark and a prospectively applied three-way centered/indeterminate/lateralized classification would further strengthen this distinction in future work.

A related direction for future work, beyond the scope of the present analysis, would be to evaluate maxillary sinus mucosal thickening in this population—its severity, and whether it extends to one or multiple paranasal sinuses—as an additional adjacent midfacial finding potentially associated with cleft laterality.

Seventh, classifying canines as erupted or unerupted does not distinguish physiological developmental non-eruption from pathological impaction. This distinction is particularly relevant in younger patients and would require longitudinal clinical information or standardized developmental criteria, which were unavailable in this retrospective dataset.

Despite these limitations, the study provides reproducible CBCT measurements and identifies significant associations between cleft laterality and the positions of the nasopalatine canal and nasal septum. These findings warrant confirmation in larger, multicenter studies using standardized imaging protocols and longitudinal assessment.

## 5. Conclusions

This retrospective CBCT study found no significant association between cleft laterality and dentoalveolar status—lateral incisor presence, root resorption, canine eruption, or cleft width—beyond the expected relationship between canine eruption and age; given the modest sample size, these findings should be regarded as inconclusive rather than as evidence of no true association. By contrast, both nasopalatine canal position and nasal septal deviation were significantly and reciprocally associated with cleft laterality. Because these two structures are anatomically distinct from the alveolar cleft itself, this dissociation supports characterizing cleft lip and palate as a regional craniofacial disturbance of the anterior nasomaxillary complex rather than an isolated alveolar defect, with potential relevance for pre-surgical CBCT planning. These findings warrant confirmation in larger, multicenter studies using standardized imaging protocols.

## Figures and Tables

**Figure 1:**
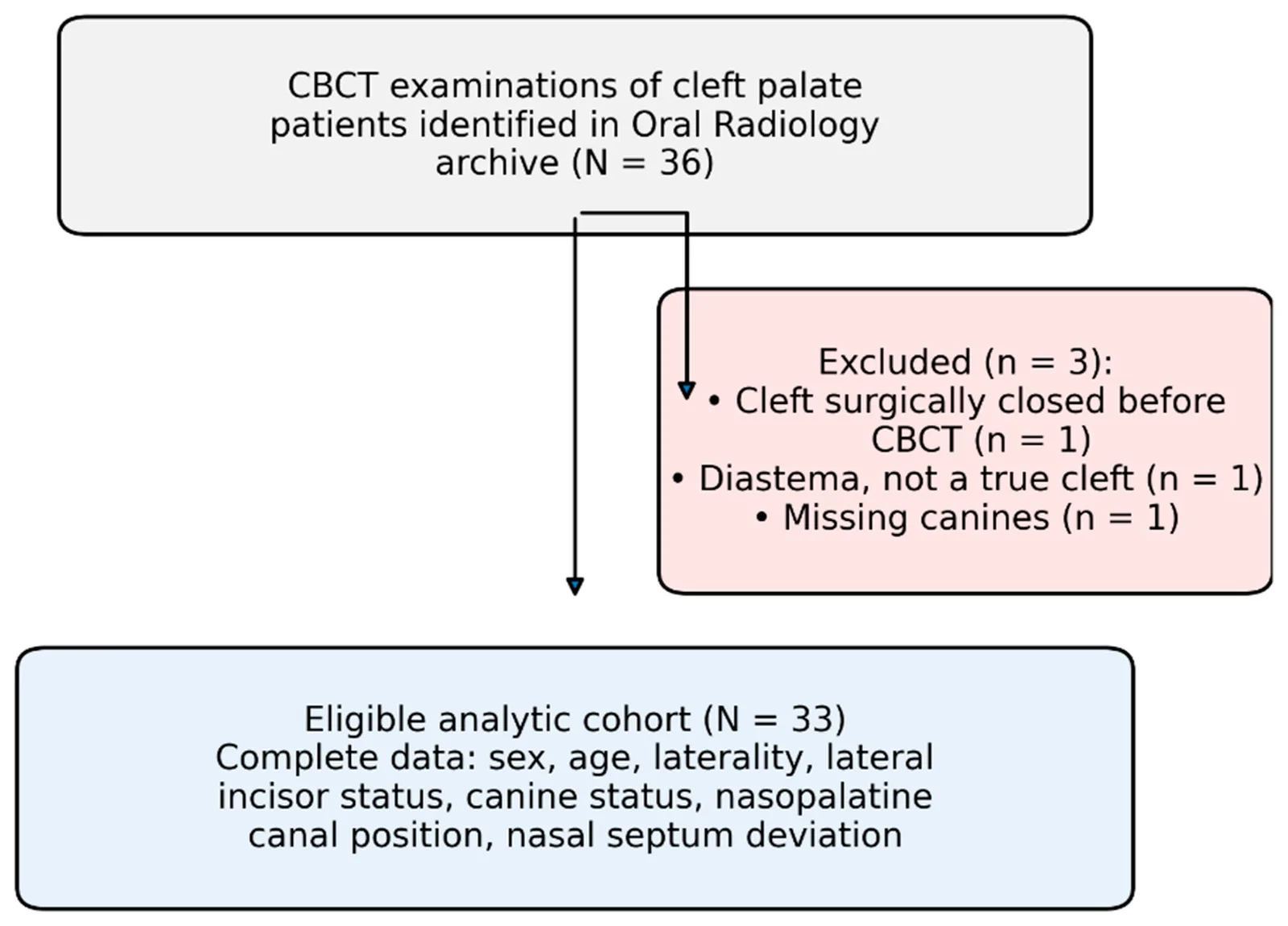
Case selection flow diagram: examinations identified, reasons for exclusion, and the resulting eligible analytic cohort.

**Figure 2:**
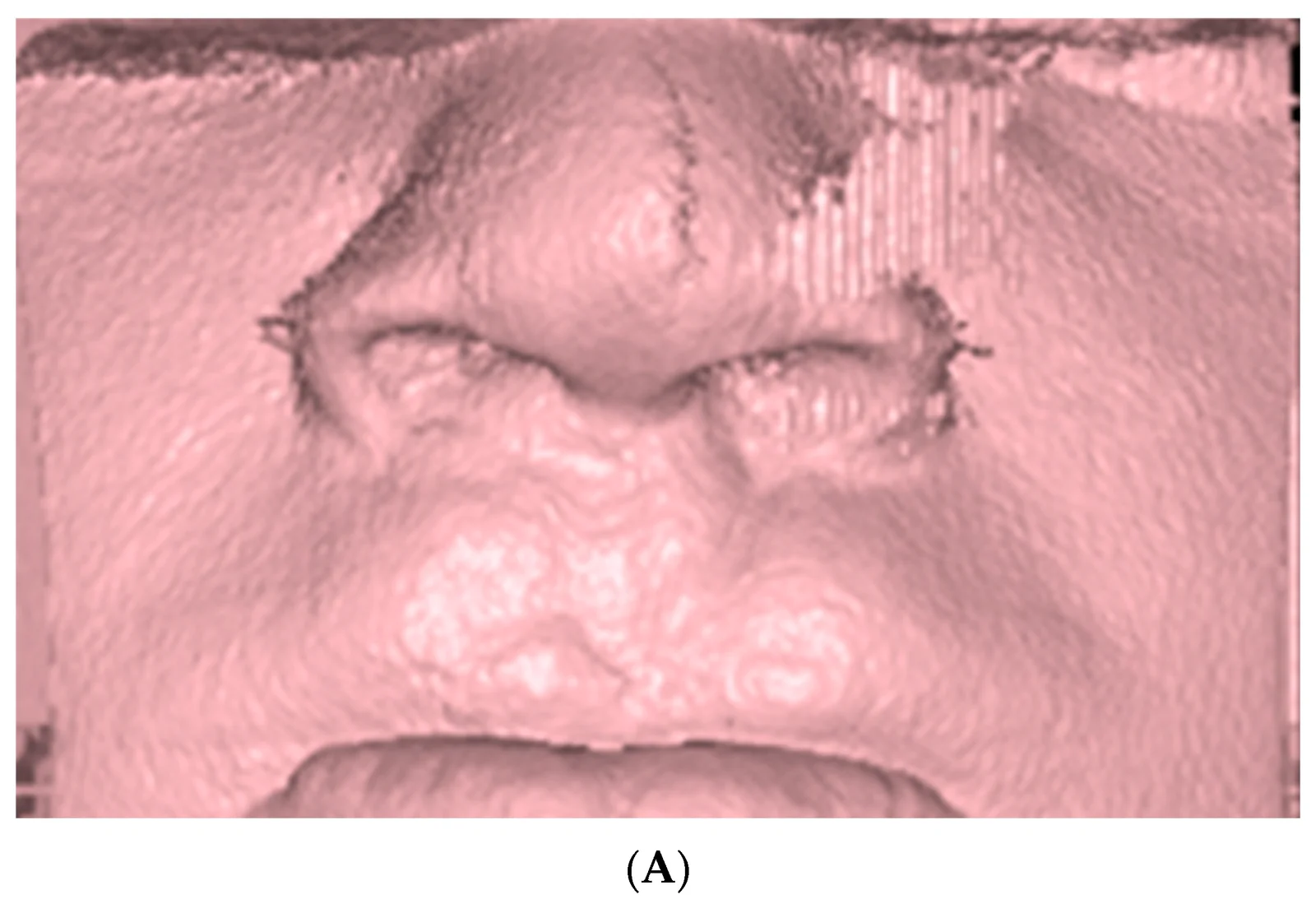

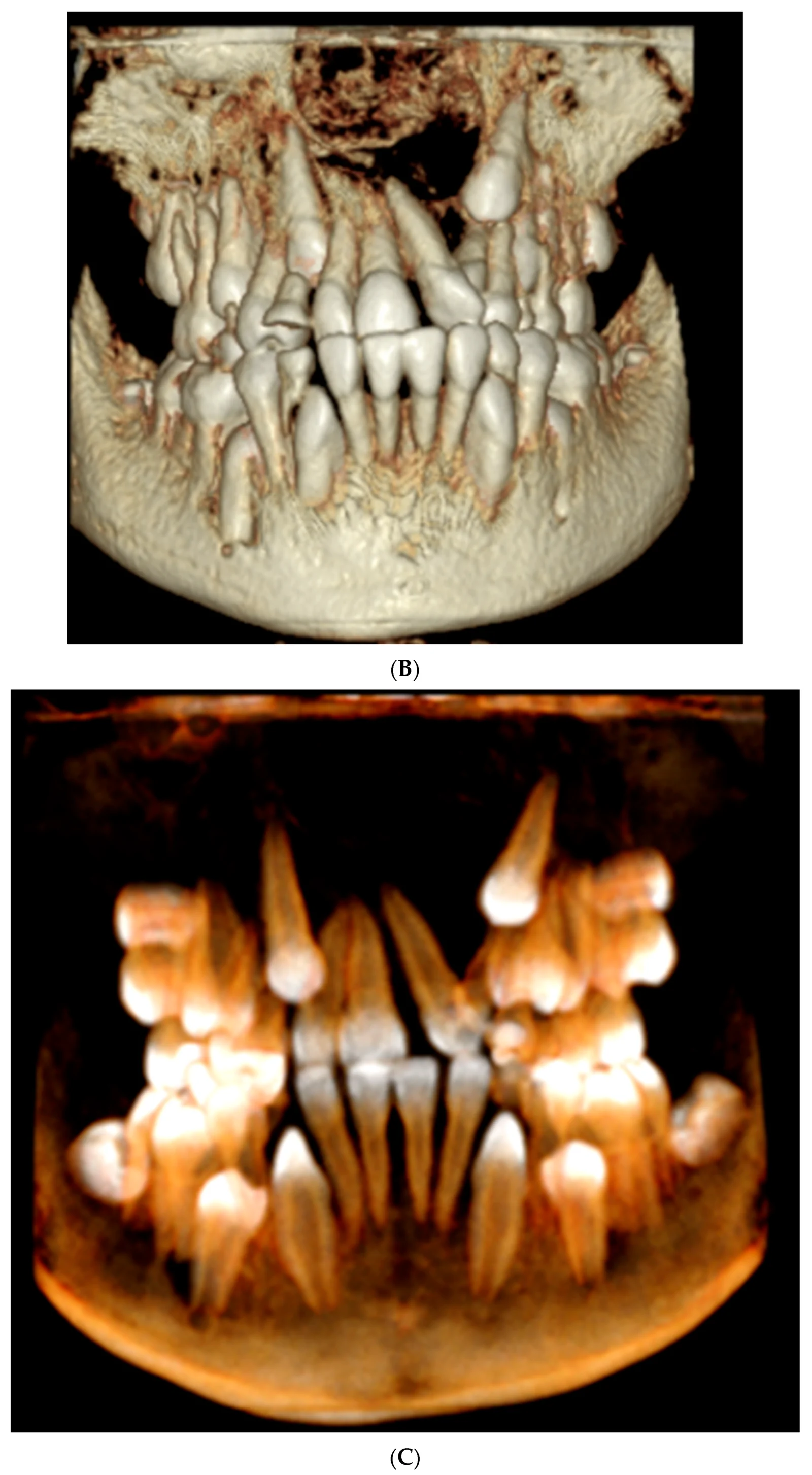

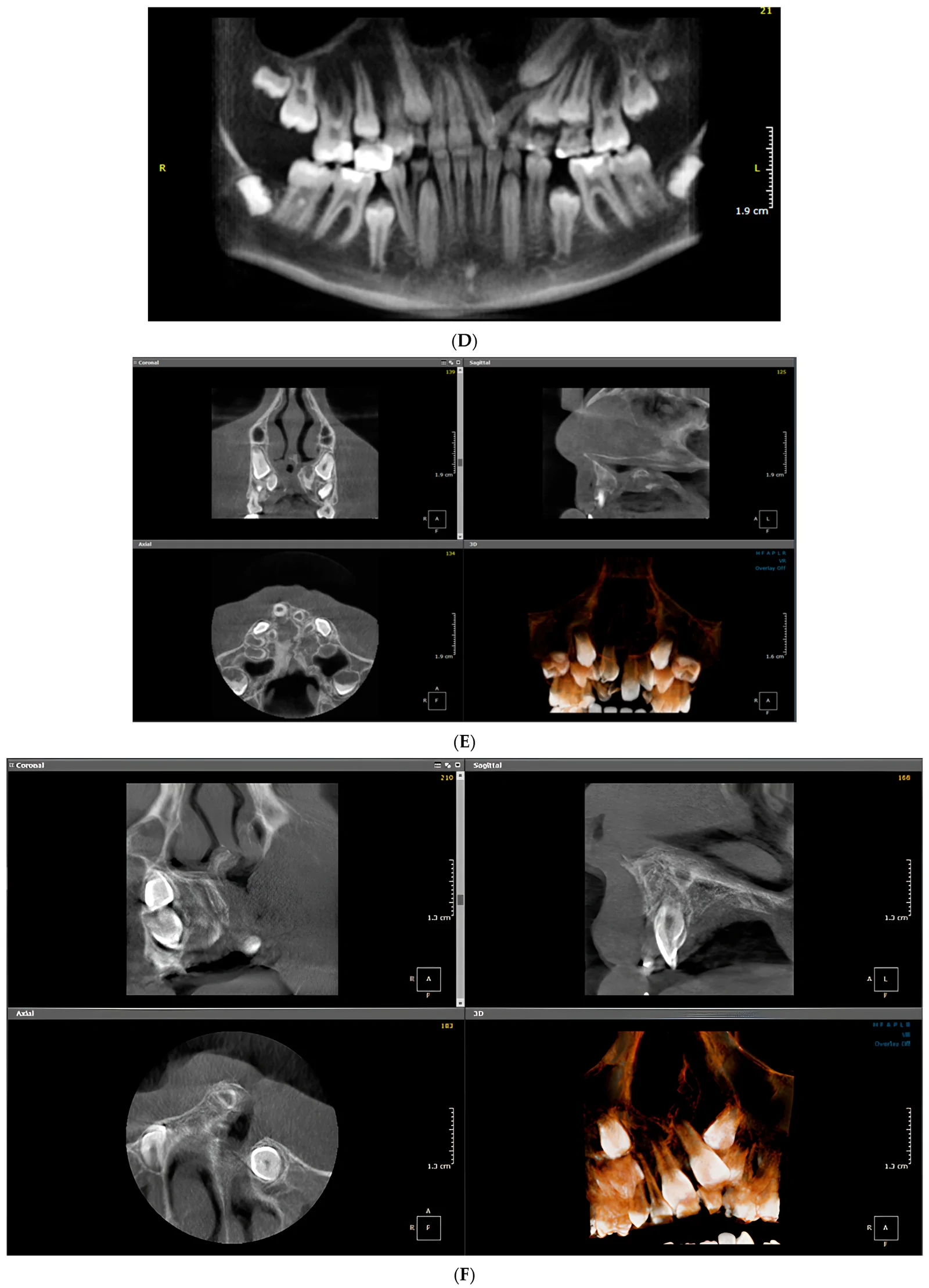

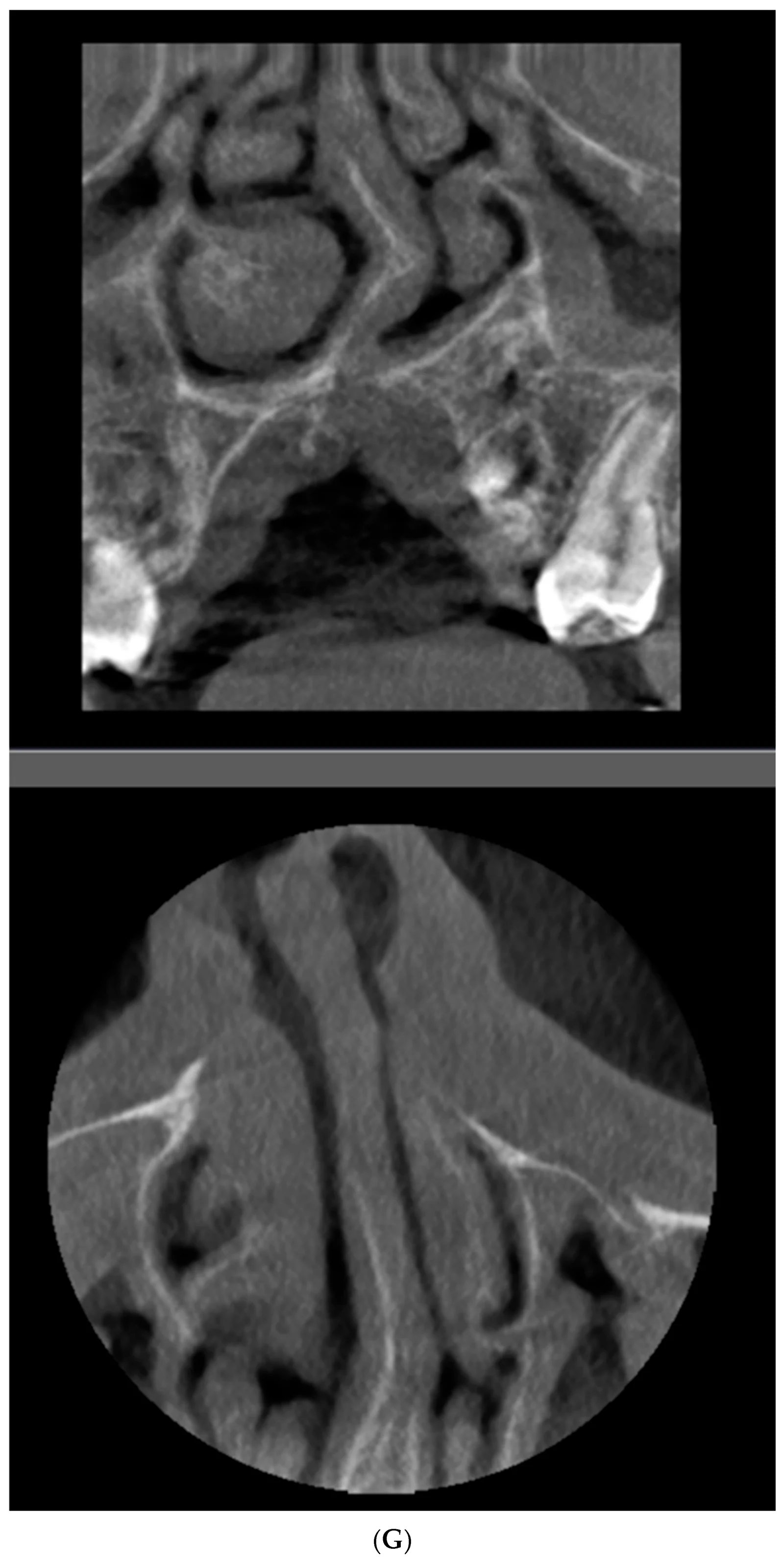
Representative CBCT reconstructions, multiplanar views, and panoramic reconstruction used for image assessment. (**A**) Three-dimensional (3D) volume-rendered soft tissue reconstruction. (**B**) 3D volume-rendered bone reconstruction. (**C**) 3D volume-rendered dentition (teeth density) reconstruction. (**D**) Panoramic reconstruction demonstrating left-sided cleft palate. (**E**) MPR view illustrating left-sided cleft palate. (**F**) MPR views demonstrating left-sided cleft palate with nasal septum deviation to the left and the nasopalatine canal located to the right of the midline. (**G**) Cropped coronal and axial MPR views showing the nasal septal deviation to the left.

**Figure 3:**
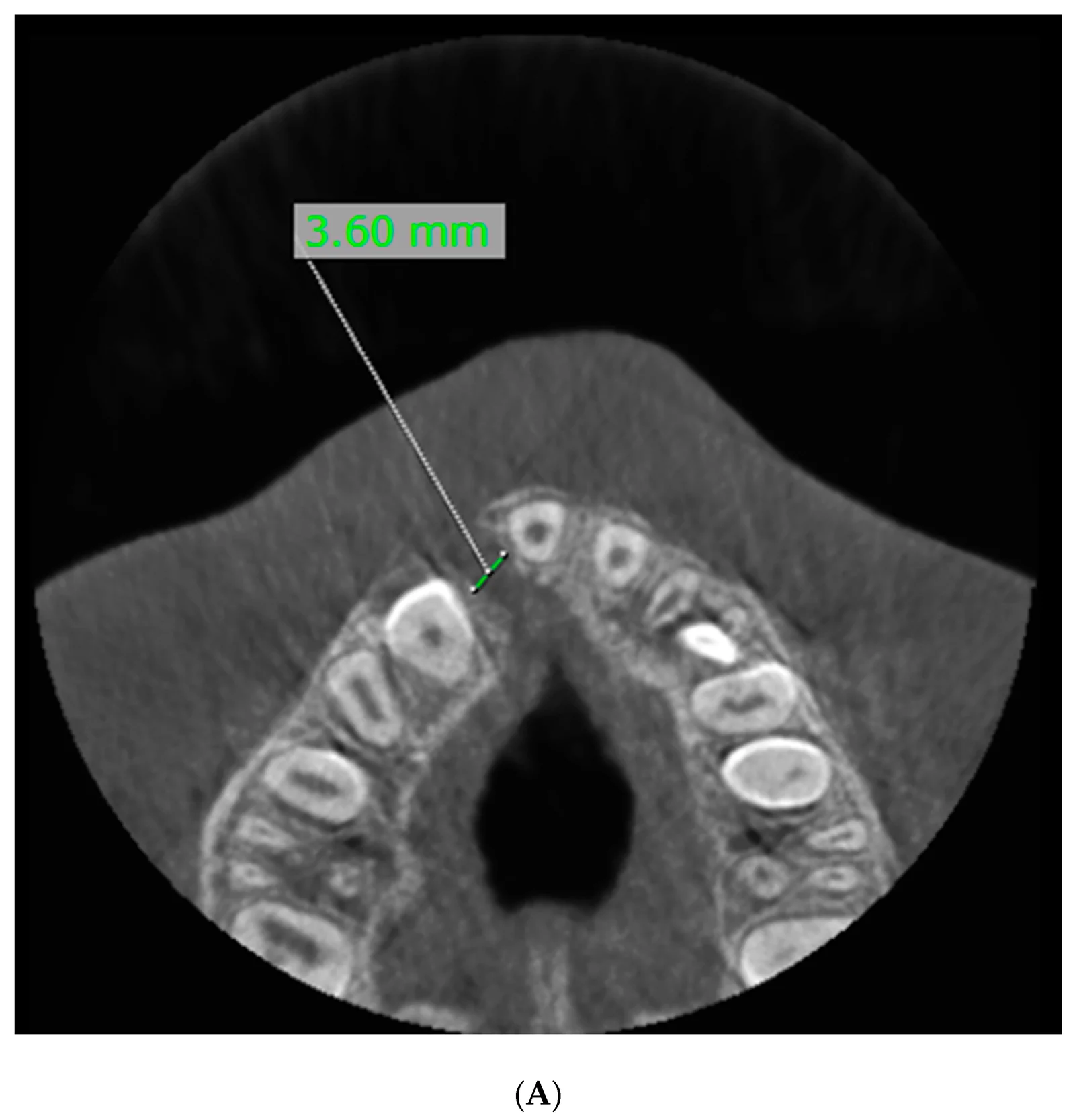

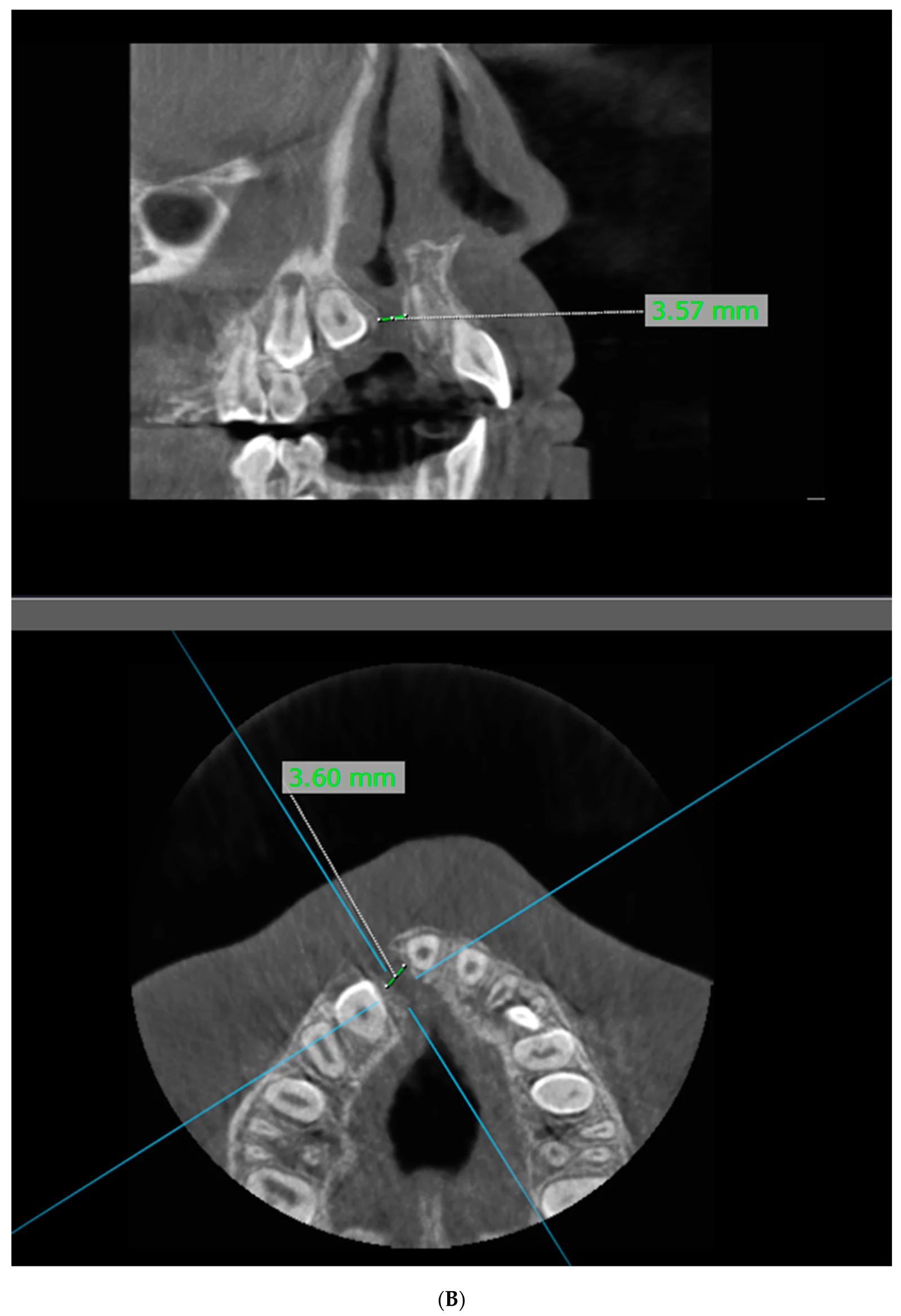
Representative CBCT images demonstrating cleft width measurement in a unilateral (right-sided) cleft case. (**A**) Axial CBCT view showing the cleft width measurement. (**B**) Corrected coronal CBCT view of the same case, with the corresponding axial reference line and cleft width measurement. The blue lines are cross-reference lines from the imaging software, indicating the orientation of the corrected cor-onal plane on the axial image.

**Figure 4:**
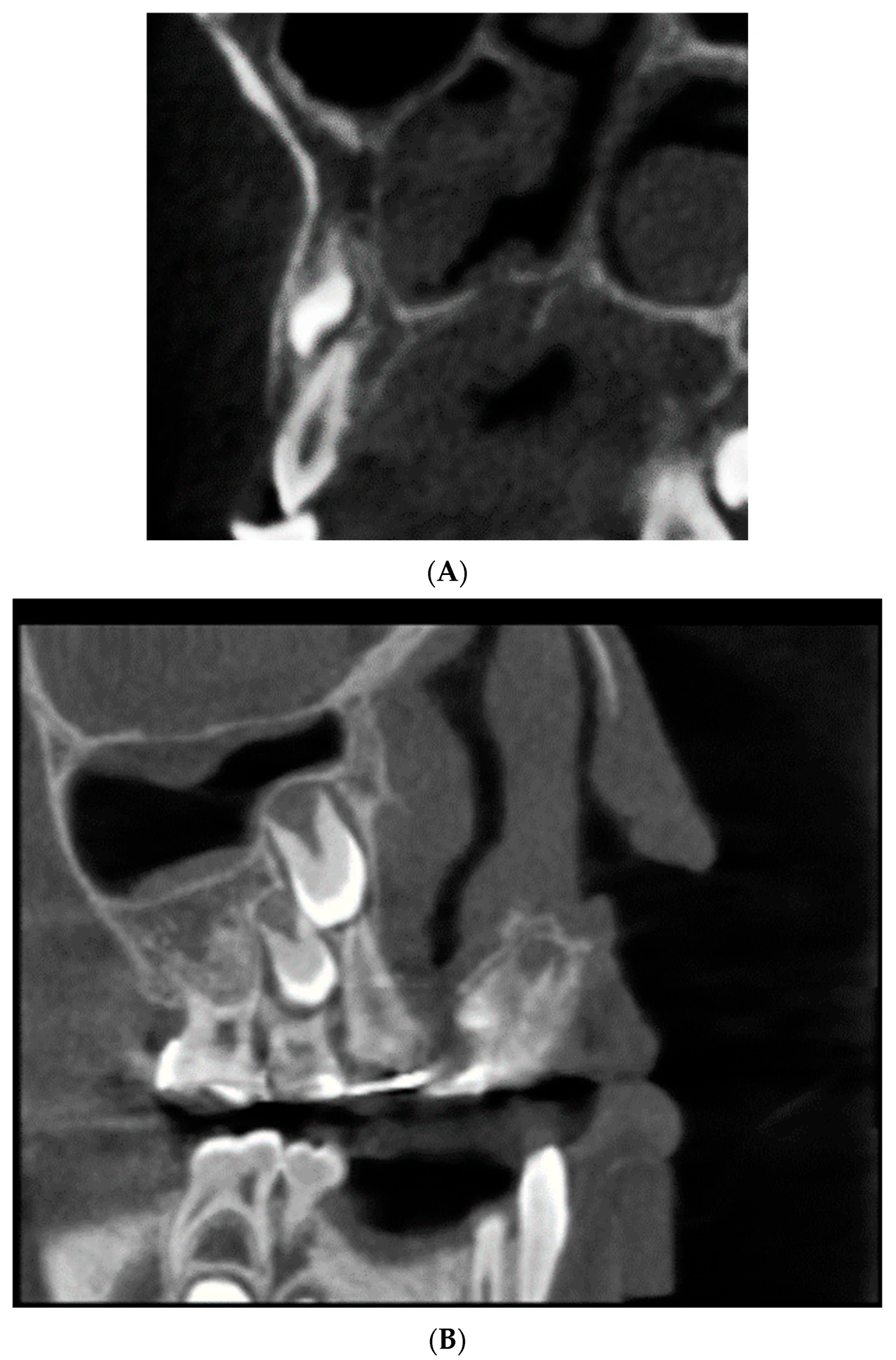

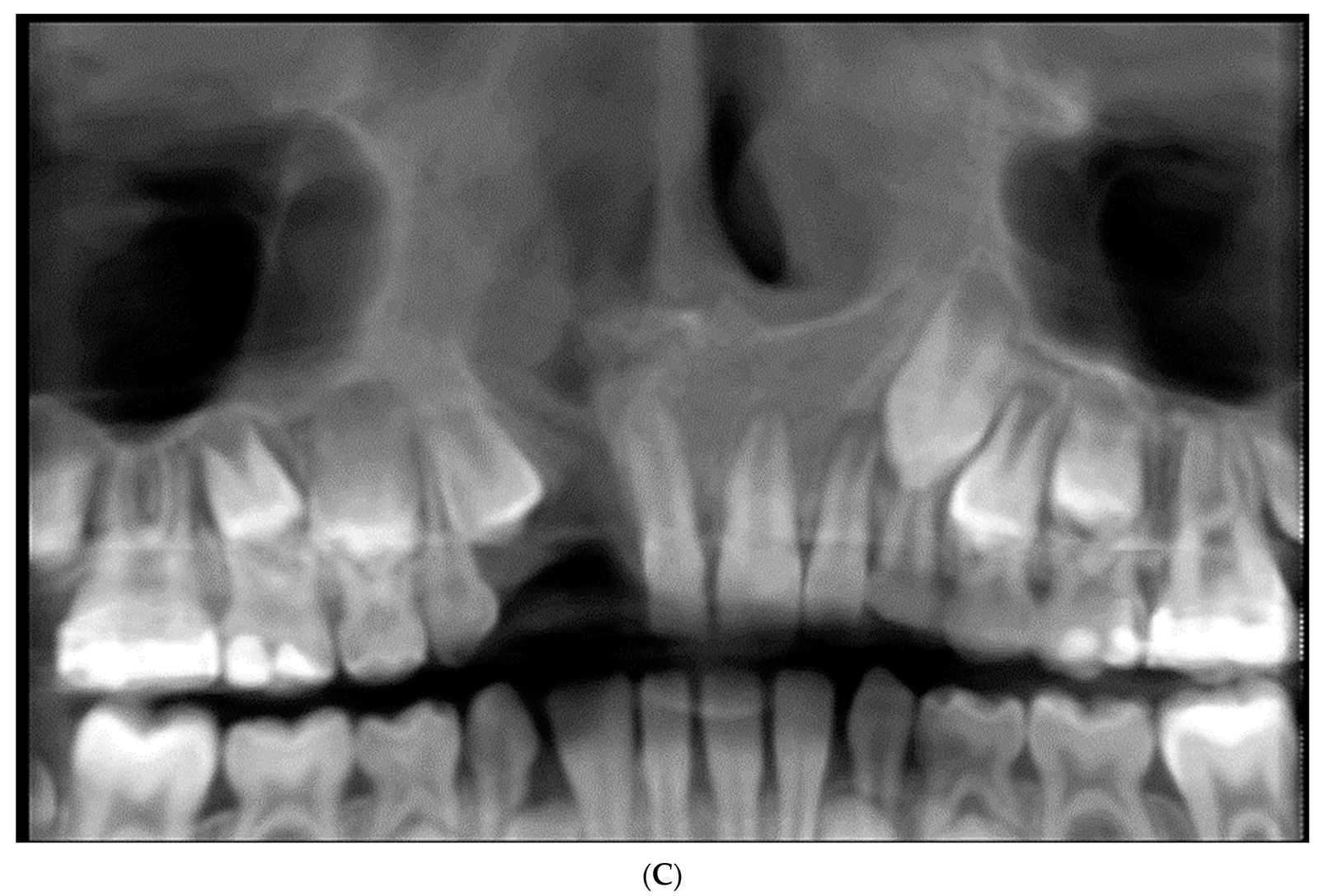
Representative CBCT images demonstrating the status of the maxillary lateral incisor adjacent to the cleft. (**A**) Corrected coronal CBCT view showing a lateral incisor without root resorption. (**B**) Corrected coronal CBCT view showing apical root resorption of the lateral incisor. (**C**) Panoramic reconstruction demonstrating an absent maxillary lateral incisor on the right (cleft) side.

**Figure 5:**
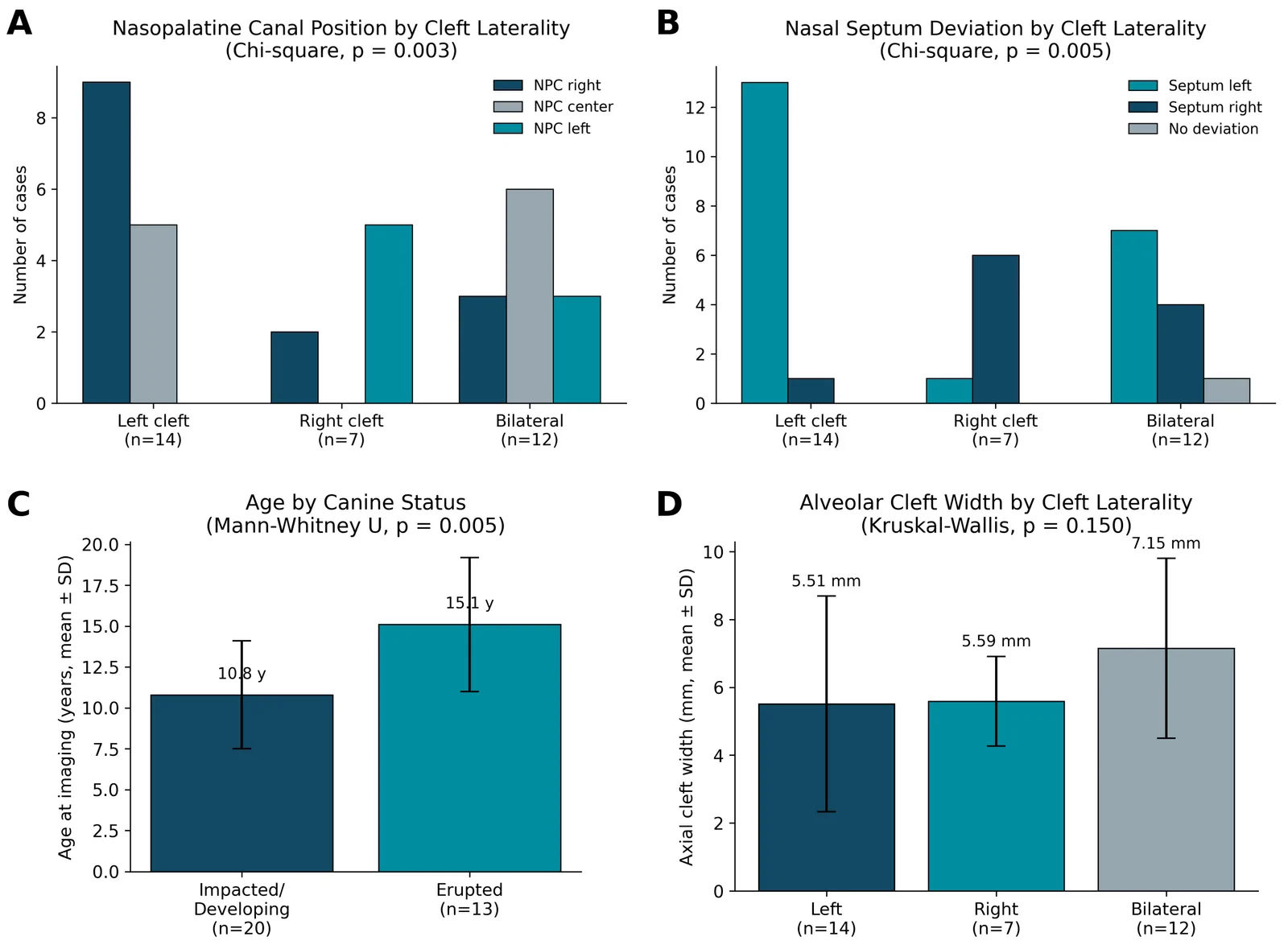
Primary outcome comparisons by cleft laterality. (**A**) Nasopalatine canal position differed significantly by cleft laterality (chi-square, *p* = 0.003). (**B**) Nasal septal deviation differed significantly by cleft laterality (chi-square, *p* = 0.005), in a pattern reciprocal to (**A**). (**C**) Age at imaging differed significantly by canine eruption status (Mann–Whitney U, *p* = 0.005). (**D**) Alveolar cleft width did not differ significantly by cleft laterality (Kruskal–Wallis, *p* = 0.150), shown for comparison. Error bars represent standard deviation; n values are shown per group.

**Table 1:** Sample characteristics of the study cohort (*n* = 33).

Characteristic	*n*	%
Female	17	51.5
Male	16	48.5
Age 7–8 years	6	18.2
Age ≥9 years	27	81.8
Unilateral left cleft	14	42.4
Bilateral cleft	12	36.4
Unilateral right cleft	7	21.2

**Table 2:** Maxillary lateral incisor status by tooth (*n* = 33).

Status	Tooth #12, *n* (%)	Tooth #22, *n* (%)
Present without root resorption	18 (54.5)	15 (45.5)
Present with apical root resorption	2 (6.1)	3 (9.1)
Absent	13 (39.4)	15 (45.5)

**Table 3:** Maxillary canine eruption status by tooth (*n* = 33).

Status	Tooth #13, *n* (%)	Tooth #23, *n* (%)
Unerupted	20 (60.6)	19 (57.6)
Erupted	13 (39.4)	14 (42.4)

**Table 4:** Descriptive relationship between lateral incisor and canine status, both sides pooled.

Lateral Incisor Status	Canine Erupted	Canine Unerupted	Total
Absent	12	16	28
Present	15	23	38
Total	27	39	66

**Table 5:** Apical root resorption among present lateral incisors (*n* = 38).

Resorption Status	*n*	%
No root resorption	33	86.8
Apical root resorption	5	13.2

**Table 6:** Patient-level lateral incisor status (*n* = 33).

Status	*n*	%
Both present	18	54.5
Both absent	13	39.4
One absent, one present	2	6.1

**Table 7:** Age and sex according to presence of at least one absent lateral incisor.

Variable	At Least One Absent (*n* = 15)	Both Present (*n* = 18)	Test	*p*-Value
Age, mean ± SD (years)	12.1 ± 3.7	12.8 ± 4.5	Mann–Whitney U = 124.0	0.703
Female, *n* (%)	9 (52.9)	8 (47.1)	Fisher’s exact	0.491
Male, *n* (%)	6 (37.5)	10 (62.5)		

**Table 8:** Summary of supplementary associations.

Comparison	Unit of Analysis	Statistical Test	Statistic	*p*-Value
Cleft side vs. lateral incisor status	Tooth-side	χ^2^	0.048	0.827
Cleft side vs. root resorption	Present teeth	Fisher’s exact	—	0.144
Cleft side vs. canine status	Tooth-side	χ^2^	0.239	0.625
Cleft laterality vs. any absent lateral incisor	Patient	χ^2^	2.510	0.285
Cleft laterality vs. any unerupted canine	Patient	χ^2^	1.736	0.420
Age vs. any absent lateral incisor	Patient	Mann–Whitney U	124.0	0.703
Age vs. root resorption	Patient	Mann–Whitney U	58.5	0.414
Age vs. any unerupted canine	Patient	Mann–Whitney U	53.5	0.005

**Table 9:** Canine status by age group.

Age Group (Years)	Unerupted, *n* (%)	Erupted, *n* (%)	Total
7–9	9 (90.0)	1 (10.0)	10
10–12	6 (75.0)	2 (25.0)	8
13–15	2 (28.6)	5 (71.4)	7
≥16	3 (37.5)	5 (62.5)	8

**Table 10:** Canine status by sex.

Sex	Unerupted, *n* (%)	Erupted, *n* (%)	Total
Female	10 (58.8)	7 (41.2)	17
Male	10 (62.5)	6 (37.5)	16

**Table 11:** Alveolar cleft measurements by side.

Measurement	*n*	Mean ± SD (mm)	Median (mm)	Range (mm)
Axial width—right	19	6.44 ± 2.70	5.65	3.00–12.50
Axial width—left	26	6.18 ± 2.78	5.95	2.20–11.50
Corrected-coronal depth—right	19	8.26 ± 5.55	6.30	3.00–27.00
Corrected-coronal depth—left	25	7.88 ± 5.21	6.30	1.30–27.00

**Table 12:** Mean patient-level axial cleft width according to cleft laterality.

Cleft Laterality	*n*	Mean ± SD (mm)
Unilateral left	14	5.51 ± 3.18
Unilateral right	7	5.59 ± 1.32
Bilateral	12	7.15 ± 2.65

Kruskal–Wallis test: H = 3.79, df = 2, *p* = 0.150.

**Table 13:** Cleft width and corrected-coronal depth correlated with sex and with the position of adjacent midline structures.

Comparison	Test	Statistic	*p*-Value
Axial width vs. sex	Mann–Whitney U	U = 114.5	0.449
Axial width vs. nasopalatine canal position (3 groups)	Kruskal–Wallis	H = 7.44	0.024
Axial width vs. nasal septal deviation	Kruskal–Wallis	H = 0.13	0.721
Corrected-coronal depth vs. sex	Mann–Whitney U	U = 116.5	0.494
Corrected-coronal depth vs. nasopalatine canal position (3 groups)	Kruskal–Wallis	H = 2.39	0.302
Corrected-coronal depth vs. nasal septal deviation	Kruskal–Wallis	H = 0.05	0.827

*Mann–Whitney U and Kruskal–Wallis H tests; alpha = 0.05. The width–canal-position association is likely confounded by the shared association of both cleft width and a centrally positioned canal with bilateral laterality (Discussion), and should not be interpreted as an independent effect.*

**Table 14:** Overall prevalence of nasopalatine canal position and nasal septal deviation (*n* = 33).

Finding	*n*	%
Nasopalatine canal—right	14	42.4
Nasopalatine canal—center	11	33.3
Nasopalatine canal—left	8	24.2
Nasal septum—left	21	63.6
Nasal septum—right	11	33.3
Nasal septum—no deviation	1	3.0

**Table 15:** Nasopalatine canal position according to cleft laterality.

Cleft Laterality	Canal Right	Canal Center	Canal Left	Total
Unilateral left	9	5	0	14
Unilateral right	2	0	5	7
Bilateral	3	6	3	12

Pearson χ^2^ = 15.94, df = 4, *p* = 0.003; Fisher–Freeman–Halton exact *p* = 0.002.

**Table 16:** Nasal septal deviation according to cleft laterality.

Cleft Laterality	Septum Left	Septum Right	No Deviation	Total
Unilateral left	13	1	0	14
Unilateral right	1	6	0	7
Bilateral	7	4	1	12

Pearson χ^2^ = 15.00, df = 4, *p* = 0.005; Fisher–Freeman–Halton exact *p* = 0.001.

## Data Availability

The data presented in this study are available on request from the corresponding author due to institutional and patient-privacy restrictions on the retrospective clinical imaging archive.

## Supplementary Materials

The following supporting information can be downloaded at https://www.mdpi.com/article/10.3390/diagnostics16193140/s1. File S1: STROBE Checklist for Cross-Sectional Studies.

## Abbreviations

The following abbreviations are used in this manuscript: CBCT, cone beam computed tomography; ICC, intraclass correlation coefficient; IPDTOC, International Perinatal Database of Typical Oral Clefts; KAUDH, King Abdulaziz University Dental Hospital; MPR, multiplanar reconstruction; WHO, World Health Organization.
