# Supplementary material for: Influence of Cleft Laterality on Cleft Width, Canine and Lateral Incisor Status, and Adjacent Midfacial Midline Structures: A Retrospective CBCT Analysis
Source: Diagnostics (Basel). 2026 Sep 27;16(19):3140. doi: 10.3390/diagnostics16193140 (PMC13663961; doi:10.3390/diagnostics16193140)
Supplement: Supplementary file 1 [file diagnostics-16-03140-s001.zip › diagnostics-4572461-supplementary-file S1.pdf]

# STROBE Statement — Checklist of Items

*Cross-sectional studies*

**Manuscript:** “Influence of Cleft Laterality on Cleft Width, Canine and Lateral Incisor Status, and Adjacent Midfacial Midline Structures: A Retrospective CBCT Analysis”

**Author:** Hanadi Sabban

**Note:** *Items shaded in orange are only partially reported in the current manuscript; a brief note follows each explaining the gap and, where applicable, what remains to be added.*

| Item No | Recommendation | Reported on page/section |
|---|---|---|
| **Title and abstract** | | |
| 1a | Indicate the study's design with a commonly used term in the title or the abstract. | Title: “…A Retrospective CBCT Analysis.” Methods, Section 2.1: study explicitly labeled “retrospective cross-sectional.” |
| 1b | Provide in the abstract an informative and balanced summary of what was done and what was found. | Abstract (structured: Background/Objectives, Methods, Results, Conclusions). |
| **Introduction** | | |
| 2 | Explain the scientific background and rationale for the investigation. | Introduction, paragraphs 1–4 (background) and closing paragraph (rationale/novelty statement). |
| 3 | State specific objectives, including any prespecified hypotheses. | Objectives (3 items) and Hypotheses (H0, H1), end of Introduction. |
| **Methods** | | |
| 4 | Present key elements of study design early in the paper. | Section 2.1 (Study Design and Setting). |
| 5 | Describe the setting, locations, and relevant dates, including periods of recruitment, exposure, follow-up, and data collection. | Section 2.1 (setting: KAUDH, Jeddah) and Section 2.2: archive searched for examinations acquired between August 2024 and June 2026. |
| 6a | Give the eligibility criteria, and the sources and methods of selection of participants. | Section 2.2 (Case Selection): archive search source, and explicit Inclusion/Exclusion criteria paragraph. |
| 7 | Clearly define all outcomes, exposures, predictors, potential confounders, and effect modifiers. Give diagnostic criteria, if applicable. | Sections 2.3–2.5 (variable definitions: lateral incisor status, canine status, cleft width, canal position, septal deviation). |

| | | |
|---|---|---|
| 8 | For each variable of interest, give sources of data and details of methods of assessment. Describe comparability of assessment methods if more than one group. | Sections 2.3–2.5 (CBCT protocol, OnDemand3D software, single examiner). |
| 9 | Describe any efforts to address potential sources of bias. | Section 2.6 (blinded intra-examiner re-read, Cohen's κ); data-verification sentence in Section 2.6; Limitations (Section 4) discusses residual bias sources. |
| 10 | Explain how the study size was arrived at. | Section 2.2: new sentence — no a priori sample-size calculation; all eligible archived examinations over the study period were included. |
| 11 | Explain how quantitative variables were handled in the analyses, including groupings. | Section 2.7 (cleft width averaged per patient; age sensitivity cut-off at 7–8 years). |
| 12a | Describe all statistical methods, including those used to control for confounding. | Section 2.7 (Fisher's exact, chi-square, Fisher–Freeman–Halton, Kruskal–Wallis, Mann–Whitney U, Spearman). |
| 12b | Describe any methods used to examine subgroups and interactions. | Section 2.7: unit-of-analysis justification (tooth-side vs. patient-level) and per-side/per-laterality subgroup comparisons. |
| 12c | Explain how missing data were addressed. | Reported per-analysis as available-N for each variable (e.g., "available for 45 cleft-side observations"); no single dedicated missing-data policy sentence — see note below. |
| 12d | If applicable, describe analytical methods taking account of sampling strategy. | Not applicable — consecutive archive sample, no complex sampling design. |
| 12e | Describe any sensitivity analyses. | Section 2.7 and Section 3.5 (sensitivity analysis excluding the six youngest patients). |
| **Results** | | |
| 13a | Report numbers of individuals at each stage of the study. | Section 3.1 and Figure 1 (flow diagram: 36 identified → 3 excluded → 33 analyzed). |
| 13b | Give reasons for non-participation at each stage. | Section 2.2 and Figure 1 (three specific exclusion reasons given). |
| 13c | Consider use of a flow diagram. | Figure 1 (case-selection flow diagram). |
| 14a | Give characteristics of study participants and information on exposures and potential confounders. | Section 3.1, Table 1 (sex, age, cleft laterality); Figure 5 panels A–D (sample and outcome distributions). |
| 14b | Indicate number of participants with missing data for each variable of interest. | Available-N reported per variable throughout Section 3 (e.g., 38 of 66 tooth-sides for lateral incisor "present" analyses). |
| 15 | Report numbers of outcome events or summary measures. | Sections 3.2–3.4 (counts and percentages for each outcome). |

| | | |
|---|---|---|
| 16a | Give unadjusted estimates and, if applicable, confounder-adjusted estimates and their precision (e.g., 95% CI). | Sections 3.2–3.4 (exact p-values throughout; 95% CI reported for ICC; κ reported without a CI — see note below). |
| 16b | Report category boundaries when continuous variables were categorized. | Section 2.2/3.5 (7–8-year age cut-off for the sensitivity subgroup, explicitly justified). |
| 16c | If relevant, consider translating estimates of relative risk into absolute risk. | Not applicable — no relative-risk estimates were calculated in this cross-sectional comparison. |
| 17 | Report other analyses done — subgroups, interactions, sensitivity analyses. | Section 3.5 (sensitivity analysis excluding youngest patients, reported for all significant findings). |
| **Discussion** | | |
| 18 | Summarize key results with reference to study objectives. | Discussion opening paragraph and Conclusion (Section 5), organized by the three study objectives. |
| 19 | Discuss limitations, taking into account sources of potential bias or imprecision. | Section 4, Study Limitations (seven numbered items, including sample size, midline-reference circularity, and categorical reliability). |
| 20 | Give a cautious overall interpretation considering objectives, limitations, and other evidence. | Discussion (comparison with prior literature) and Conclusion (explicitly cautious framing of non-significant findings as inconclusive). |
| 21 | Discuss the generalisability (external validity) of the study results. | Partly addressed via the single-center, retrospective design noted in Limitations; no dedicated generalizability sentence — see note below. |
| **Other information** | | |
| 22 | Give the source of funding and the role of the funders. | Funding section (Deanship of Scientific Research, King Abdulaziz University, grant IPP: 976-165-2026). |

## Notes on partially-reported items

**Item 12c (missing data):** Missingness is reported transparently as an available-N for each specific analysis, but there is no single dedicated sentence stating the overall missing-data policy (e.g., complete-case analysis). Recommend one sentence in Section 2.7, such as: “Analyses used all available data for each variable; cases with missing values for a given variable were excluded only from that specific analysis (complete-case basis).”

**Item 16a (precision for κ):** Cohen's κ values (0.86, 0.88) are reported as point estimates without a 95% confidence interval. Recommend adding CIs if the underlying re-read data are still available (most statistical software reports these alongside κ).

**Item 21 (generalisability):** The single-center, retrospective nature is noted in Limitations, but there is no dedicated sentence explicitly discussing how well these findings might generalize to other populations or imaging protocols. Recommend one sentence in Discussion or Limitations addressing this directly.
